# Enhanced Magnetic Hyperthermia Performance of Zinc Ferrite Nanoparticles under a Parallel and a Transverse Bias DC Magnetic Field

**DOI:** 10.3390/nano12203578

**Published:** 2022-10-12

**Authors:** Constantin Mihai Lucaciu, Stefan Nitica, Ionel Fizesan, Lorena Filip, Liviu Bilteanu, Cristian Iacovita

**Affiliations:** 1 Department of Pharmaceutical Physics-Biophysics, Faculty of Pharmacy, Iuliu Hatieganu University of Medicine and Pharmacy, 6 Pasteur St., 400349 Cluj-Napoca, Romania; 2 Department of Toxicology, Faculty of Pharmacy, Iuliu Hațieganu University of Medicine and Pharmacy, 6A Pasteur St., 400349 Cluj-Napoca, Romania; 3 Department of Bromatology, Hygiene, Nutrition, Iuliu Haţieganu University of Medicine and Pharmacy, 6 Pasteur St., 400349 Cluj-Napoca, Romania; 4 Department Preclinical Sciences, Faculty of Veterinary Medicine, University of Agronomic Sciences and Veterinary Medicine of Bucharest, 105 Splaiul Independentei, 050097 Bucharest, Romania; 5 Molecular Nanotechnology Laboratory, National Institute for Research and Development in Microtechnologies, 126A Erou Iancu Nicolae St., 077190 Bucharest, Romania

**Keywords:** magnetic hyperthermia, magnetic nanoparticles, saturation of the SAR, SAR dependence on the concentration, chains formation, static DC magnetic field, immobilization of magnetic nanoparticles, hyperthermia coercive field

## Abstract

The collective organization of magnetic nanoparticles (MNPs) influences significantly their hyperthermic properties, relevant for their in vitro and in vivo applications. We report a systematic investigation of the effects of the concentration and the static bias direct current (DC) magnetic field superposed over the alternating magnetic field (AMF), both in a parallel and perpendicular configuration, on the specific absorption rate (SAR) by using zinc ferrite MNPs. The nonmonotonic dependence of the SAR on the concentration, with a maximum at very small concentrations (c ≤ 0.1 mgFe/mL), followed by a minimum at 0.25 mgFe/mL, and the second maximum of 3.3 kW/gFe at around 1 mgFe/mL, was explained by the passage of the MNPs from a single particle behavior to a collective one and the role of the dipolar interactions. By superposing a static 10 kA/m bias DC field on the AMF we obtained an increase in the SAR for both parallel and perpendicular orientations, up to 4285 W/g_Fe_ and 4070 W/g_Fe_, respectively. To the best of our knowledge, this is the first experimental proof of a significant enhancement of the SAR produced by a perpendicular DC field. The effect of the DC field to increase the SAR is accompanied by an increase in the hyperthermia coercive field (H_cHyp_) for both configurations. No enhancement of the DC fields was noticed for the MNPs immobilized in a solid matrix but the DC field increases the H_cHyp_ only in the parallel configuration. This translates into a higher SAR value for the perpendicular configuration as compared to the parallel configuration. These results have practical applications for magnetic hyperthermia.

## 1. Introduction

In recent decades, an exponential increase in the research field of magnetic nanoparticle (MNP) applications has been recorded in various domains of knowledge, especially in the biomedical field [1]. Among the many applications of MNPs in the biomedical field, magnetic hyperthermia (MH) has been intensively investigated due to its enormous potential in cancer therapy [2,3,4]. Although the first clinical trial was initiated over a decade ago [5], the method is approved in Europe for treating brain tumors (glioblastoma multiforme) and is routinely applied to eligible patients independently of other clinical trials. However, no widespread adoption of this method has been observed in current clinical practice. Numerous studies have been initiated to improve the hyperthermic properties of MNPs and to make them capable of dissipating sufficient heat under the action of an alternating magnetic field (AMF) to raise the temperature of the target tissue either for inducing thermal ablation or for its sensitization so that in conjunction with radio- or/and chemotherapy, to obtain tumor eradication. Last but not least, the therapeutic temperature must be achieved in safe conditions for the patient, limiting thus the doses of MNPs used and the amplitude (H_max_) and frequency (f) of the AMF [6,7].

Due to the large number of investigations performed, the MH properties of individual MNPs and the parameters on which they depend (size, size dispersivity, shape, saturation magnetization–M_s_, coercive field–H_c_, anisotropy constant–K, and others) when the MNPs are fixed and magnetically independent, have been largely clarified [8,9]. However, the roles of the different parameters in MH are still under investigation. For example, it was demonstrated experimentally that the shape anisotropy plays an important role in the heating performances of MNPs with anisotropic shapes, such as nano-octopods, cubes, and nanorods performing better than their spherical counterparts [10,11,12]. However, the shape anisotropy increase in the heating is strongly influenced by the size of the MNPs, with small cubes having better performances as compared to spheres but larger spheres heating better than larger cubes [13]. Another line of research for improving MH is to create new structures by combining the different core-shell bimagnetic structures [14], or the hybrid ones for combinatorial therapies [15,16].

Nevertheless, when the MNPs are free to move and present strong magnetic interactions, the complexity increases significantly, as the MNPs self-organize. The properties of the self-organized assemblies are meaningfully different from the case of single-nanoparticles (NPs).

The in vitro and in vivo collective behavior of MNPs revealed that their hyperthermic properties are significantly lower than those in aqueous suspensions, which is explained by their intracellular agglomeration or aggregation [17,18,19]. Both the theoretical and experimental reports have shown that dipole-dipole interaction can lead either to a chain formation or a random agglomeration into spherical-like clusters, depending on the MNPs’ anisotropy, size, concentration, and H_max_. Chain structures exhibit an increased uniaxial anisotropy and a subsequent increase in the heating performance, while random structures exhibit reduced mobility and a magnetic moment, thus reducing their heat release [19]. The interest in such studies was also stimulated by the fact that magnetosomes, MNPs secreted by magnetotactic bacteria, have remarkable hyperthermic properties, often surpassing those of their synthetic counterparts [20]. The magnetosomes’ high heating capability was explained by their high chemical purity (they are made of magnetite) as well as by their chain organization [6,20]. The fact that the chain organization of MNPs increases their heating properties was explained theoretically by various approaches such as the Monte-Carlo simulations [21], the Landau–Lifshitz–Gilbert equation [22], or the Brownian dynamics simulations [23], by an increase in the squareness of their dynamic hysteresis loops and therefore of the loop area. The hysteresis loop area provides the heat released during one cycle, which, multiplied by the frequency, represents the energy dissipated in unit time and the expressed per unit mass of MNPs is called the specific absorption rate (SAR) or specific loss power (SLP).

The theoretical results were also verified experimentally, mostly by experiments in which MNPs were aligned in a static direct current magnetic field (H_DC_) before their im-mobilization (gellation) in a solid matrix, revealing that MNPs aligned parallel to the AMF have a much higher heating power as compared to the random case [21,23,24,25]. Our experimental results [26,27] demonstrate that MNPs randomly immobilized in a solid matrix, reduce their SAR by almost 50% due to the blocking of their physical rotation (the Brown relaxation mechanism). However, if the MNPs are pre-aligned in a static direct current magnetic field (H_DC)_ before their immobilization in a solid matrix and exposure to the AMF, they recover their SAR values in water [28]. Moreover, we noticed that in the hyperthermia coercive field (H_chyp_), the field strength corresponding to the maximum slope in the sigmoidal SAR dependence on the H_max_ is reduced with the increasing MNP concentration in water suspensions. This observation, not detected in the case of immobilized samples, could be explained by the organization of the MNPs in chains induced by the AMF. The effect is more pronounced at higher concentrations because the MNPs are more prone to organize in chains as the concentration increases. The chain formation in water suspensions of MNPs under the influence of the AMF is supported by the recent findings of another group, showing that in large MNPs, the applied magnetic field induces an increase in the magnetic susceptibility and subsequently, an increased SAR [29]. Using time-resolved high-frequency hysteresis loops, it was demonstrated, that under the influence of the AMF, the chains are formed on a timescale strongly depending on the H_max_, ranging from 100 ms to several tens of seconds [30,31]. Moreover, it was demonstrated that both the heating power and the squareness of the dynamic hysteresis loops increase with time as the chains form progressively [31].

Another interesting approach for increasing the heating power of the MNPs, is to superpose an H_DC_ to the AMF during the hyperthermia measurements. It was experimentally demonstrated that such an approach, for MNPs in the superparamagnetic state (SP-MNPs), can increase their SAR values up to 40% when compared to the situations when only the AMF is applied [32]. However, an extra H_DC_ perpendicular to the direction of the AMF reduces the measured SAR values [33]. On the other hand, the ferromagnetic nanoparticles (F-MNPs) aligned under the H_DC_ in agar solutions of different concentrations (0.10–2.00% wt agar concentration range), behaved differently. They exhibit a significant SAR increase (up to 3-fold) in both the parallel and perpendicular configurations of the H_DC_, with respect to the AMF for the samples of a very low agar concentration (0.1% wt. agar) allowing the F-MNPs mobility [21].

Aiming at obtaining more insights into the effects of the H_DC_ on the MH performances of F-MNPs, in this paper, we report the influence on the SAR values of Zn ferrite NPs (dispersed in water or immobilized in a solid matrix) of an H_DC,_ parallel and perpendicular to the AMF. The Zn ferrite MNPs were chosen because it is well-known that the Zn substitution increases the saturation magnetization in ferrites. Moreover, we synthesized the MNPs with a relatively large size (28 nm) not surpassing the monodomain limit, possessing thus a high magnetic moment per MNP and assuring high Zeeman and dipolar interaction energies. It is also important to mention that recently, it was demonstrated that core-shell ferrite MNPs with a shell doped with Zn, exhibit an unusually high SAR [14]. As reported previously [34], these Zn ferrite MNPs exhibit very high SAR values, above 3 kW/g_Fe_, and are also nontoxic for both normal BJ and A459 cancer cell lines, up to a concentration of 0.8 mg/mL [35].

In the first step, we analyzed the concentration dependence of the Zn ferrites’ SAR, which varies nonmonotonically as a function of concentration. Through this investigation, we obtained more information about the nature of the dominant interactions at a given concentration relevant to MH. 

The application of an H_DC_ of either 10 kA/m or 20 kA/m, parallel to the AMF during the MH measurements, increases the maximum SAR values in a concentration-dependent manner, the effect being more pronounced at low concentrations. For both values of the H_DC_, the increase in the SAR is accompanied by an increase in the H_chyp_, meaning that the maximum SAR values are obtained at higher values of the amplitude of the AMF (H_max_). However, in the case of the perpendicular configuration, the H_DC_ field of 10 kA/m increases the SAR values while an H_DC_ of 20 kA/m strongly decreases the heating properties of the Zn ferrites. For the immobilized samples, we show that the pre-alignment of the MNPs in an H_DC,_ leads to an increase in the SAR, but the further application of the H_DC_ during the MH experiments does not change the SAR significantly. 

Our results are significant for future in vitro and in vivo MH applications because the SAR dependence on the concentration and the H_DC_ is of paramount importance for efficient heating of MNPs in adjuvant cancer therapies.

## 2. Materials and Methods

### 2.1. Synthesis and Physicochemical Characterization of Zinc Ferrites Nanoparticles

The zinc ferrite NPs were synthesized following a thermal decomposition method of the acetylacetonate precursors [34]. The detailed synthesis method and the physicochemical characterization techniques as, transmission electron microscopy (TEM), X-ray diffraction (XRD), dynamic light scattering (DLS), Fourier transform infra-red spectroscopy (FTIR), and vibrating sample magnetometry (VSM) are presented in our previous paper [35]. Briefly, the as-synthesized MNPs have faceted shapes (some are cubic, others polyhedral) with the main size of 28 ± 0.2 nm and a good polydispersity index which was very close to the average crystalline size (27 nm), calculated using Scherrer’s formula by the Gaussian fit of the prominent X-ray diffraction peaks (Figure S1) [35]. The energy-dispersive X-ray (EDX) maps show that the Fe and Zn elements are homogeneously distributed within the total volume. At the same time, the quantitative analysis of the EDX spectra revealed a mean value of the Zn atomic percentage around 0.4, i.e., the Zn ferrites correspond to the formula Zn_0.4_Fe_2.6_O_4_ (Figure S1) [35]. The M_s_ is around 100 emu/g at 4 K and decreases to 73 emu/g at room temperature (RT), this decrease being consistent with the literature and can be attributed to the increased spin-disorders in the surface layers of the smaller MNPs [36]. The Zn ferrites preserve the ferromagnetic character at RT, as the coercive field (H_c_) slightly decreases from 29 mT (24 kA/m) to 19 mT (15 kA/m) by increasing the temperature from 4 K to RT (Figure S1) [35]. The oxidation of the oleic acid by sodium periodate induces Zn ferrites a zeta potential of −52 mV, due to the resulting carboxyl groups [37], enabling a good colloidal stability. However, according to the DLS data, the ferromagnetic Zn ferrites have a mean hydrodynamic diameter of 70 nm in water, signifying that they stabilize in an aqueous solution in very small clusters [35]. The main physical parameters obtained from the physicochemical characterization relevant to the current study are summarized in Table 1. 

### 2.2. Magnetic Hyperthermia

For the MH experiments, an EasyHeat 0224 power supply station (Ambrell, Scottsville, NY, USA), operating at frequencies between 100–400 kHz and able to generate magnetic fields with strengths of up to 65 kA/m, has been employed. The system is equipped with an eight-turn coil with an internal diameter of 2.5 mm and a total length of 40 mm. The inductance of the coil was calculated from its geometry and the resonance frequency of the circuit in which it was introduced and was determined as 10^−6^ H. The effective frequency and voltage values on the coil were monitored with a digital oscilloscope PeakTech 1170 (PeakTech Prüf-und Messtechnik GmbH, Ahrensburg, Germany) operating up to 250 MHz, and for the specified coil was 355 kHz. The H calibration was performed by using a copper wire with a 10 mm diameter, surrounding the vial in which the samples were introduced as a magnetic probe, and measuring the induced electromotive force by using the oscilloscope, as described in detail in our previous work [26]. The samples consisted of a 0.5 mL volume of Zn ferrites suspended in water or dispersed in a solid matrix of polyethylene glycol 8000 (PEG 8K). The temperature was assessed using a fiber optic, placed in the middle of the sample, to provide the temperature values at 1-s intervals. 

The temperature changes ΔT versus the time curves presented in Figures S2 and S3 have been fitted with the Box–Lucas equation:

(1) $$\Delta T=\frac{S_{m}}{k}\;\left(1-e^{-k\left(t-t_{0}\right)}\right)$$

where the fitting parameters $S_{m}$ and k are the initial slope of the heating curves and the constant describing the cooling rate, respectively. Thus, the SAR can be calculated as:

(2) $$\mathrm{SAR}=\frac{{c\;m\;S}_{m}}{m_{\mathrm{Fe}}}\;$$

where c is the colloid-specific heat (the Zn ferrites’ contribution to the specific heat being negligible in our case) and was approximated with the c of either water or PEG 8K m = *ρ*V is the mass of colloid, taken as the product between the density and the volume. The following values were used in the calculations: for water *ρ* = 0.997 g/cm^3^, c = 4186 J/kgK; for PEG 8K: *ρ* = 1.0852 g/cm^3^, c = 2135.27 J/kg. As previously described, the iron concentration of the samples was determined using the thiocyanate assay [35]. Prior to each measurement, the liquid samples have been sonicated for 15 s to ensure a good colloidal dispersion over the entire aqueous volume. Each SAR value is a mean of a minimum of three measurements realized on three different samples.

### 2.3. Immobilization and Alignement of the Zn Ferrites in a Solid Matrix 

The Zn ferrites, at the desired concentrations, dispersed in water, were collected at the bottom of the vial by a magnet, the water was discharged, and 0.5 mL liquid PEG 8K heated at 80 °C was introduced. The samples were immediately sonicated using a Vibra-Cell™ Ultrasonic probe sonicator, model VCX 500 equipped with a tapered microtip of Ø 6 mm (Sonics&Materials, Inc., Newtown, CT 06470, USA) to assure an excellent dispersion of the Zn ferrites on the entire volume of the liquid PEG 8K. Then, some samples were placed on a vial support rack for solidification, while others were solidified in the presence of 65 kA/m H_DC_, generated by two cubic neodymium magnets with a 3 cm side length, integrated into a homemade support. The magnetic induction, measured with a Brockhaus Gaussmeter BGM 101 (Dr. Brockhaus Messtechnik GmbH & Co. KG, D-58507 Ludenscheid, Germany), is almost constant in the middle region of around 1 cm^3^, between the two magnets, according to the calibration curve provided in Figure S4.

### 2.4. Magnetic Hyperthermia under a Static DC Magnetic Field 

The samples containing the suspensions of the Zn ferrites in water or PEG 8K were placed in the middle of the eight-turn coil of the EasyHeat 0224 power supply station. At the same time, an H_DC_ was created by using two neodymium magnets with a 3 cm side length, placed above and below the coil for the parallel configuration, and on opposite sides of the coil for the perpendicular configuration (Figure S5). The distance between the two neodymium magnets was varied to obtain the H_DC_ of the desired intensity (13 cm for 10 kA/m and 18 cm for 20 kA/m) at the sample level within the eight-turn coil.

## 3. Results and Discussion

### 3.1. Magnetic Hyperthermia of Zn Ferrites Dispersed in Water 

The zinc ferrite NPs were chosen to test the effects of the H_DC_ on their MH performances, due to their higher M_s_ and high magnetic moments. The diamagnetic Zn^2+^ (d^10^) cations, once incorporated in the spinel structure of the magnetite (Fe_3_O_4_), can produce a significant enhancement of the NPs’ magnetic moment, [34,38,39,40] due to their unique tendency to occupy the tetrahedral sites in the spinel structure, forcing the trivalent Fe^3+^ (d^5^) cations to migrate to the octahedral sites by replacing the divalent Fe^2+^ cations. This scenario is valid until a certain Zn content is achieved in the spinel structure, which depends on the employed synthetic route. Our previous studies present several synthesis methods for obtaining Zn ferrites, including coprecipitation, polyol, or thermal decomposition [28,41,42]. For this paper, we opted for the high-temperature thermal decomposition method of the acetylacetonates magnetic precursors, as this method produces MNPs with a high crystallinity [16,34,35], allowing at the same time, by appropriately adjusting the temperature ramps and the magnetic precursor concentrations to control the size of the synthesized MNPs. Our goal was to obtain large MNPs exhibiting a ferromagnetic character at RT, without surpassing the monodomain limit.

**Figure 1:**
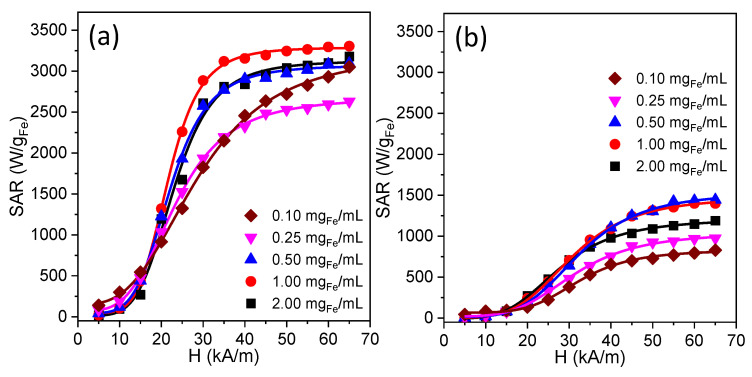
SAR dependence on the H for different concentrations of the Zn ferrites dispersed in (**a**) water and (**b**) PEG 8K. The lines are the best fits obtained by using the logistic function.

The MH properties of the Zn ferrite NPs were assessed using the AMF from 5 to 65 kA/m at 355 kHz. From our previous results [27,28], it is obvious that a complete characterization of the hyperthermic properties of MNPs involves using a broad range of H_max_ values, as the SAR dependence on the H_max_ is nonlinear and saturates at high H_max_ values. In the first step, we studied the SAR dependence of the Zn ferrite NPs, dispersed in water, on both the H_max_ and the concentration (Figure 1). For all concentrations, the SAR reaches very high values (above 3 kW/g_Fe_), indicating that the cation substitution strategy effectively increases the M_s_ and the SAR. On the other hand, it can be noticed that the SAR dependence on the H_max_ is sigmoidal, which corresponds to very low SAR values at a low H_max_, followed by a steep increase of the SAR values by increasing the H_max_ and the saturation of the SAR values mainly at the high H_max_. The saturation of the SAR values at a high H_max_ is well known for F-MNPs, while for SP-MNPs, the linear response theory [8] predicts a square dependence of the SAR on the H_max_, without saturation. This assumption is valid only for a small H_max_. In one of our previous studies [42], we demonstrated experimentally that saturation occurs also in the case of SP-MNPs and that their heating properties could be described only by considering both, a nonlinear (Langevin function type) dependence of the magnetization on the H_max_ and also taking into account the H_max_ dependences of the Néel and Brown relaxation times.

The heating abilities of F-MNPs, as in our case, could be described by the Stoner–Wolfharth theory-derived models [8]. For MNPs with uniaxial anisotropy and when the anisotropy energy is much larger than the thermal one, the magnetization presents only the two most probable positions, corresponding to the two wells in the energy landscape. Based on this model, the dynamic hysteresis curves can be numerically calculated, the area of the hysteresis loop providing the heat released by the MNPs during one cycle. However, no analytical function derived from a theoretical model is available yet in the literature, to correlate the magnetic characteristics of the MNPs to their SAR performance. In the case of a pure Stoner–Wohlfarth model, only two orientations are possible for the magnetization, and no thermal activation is taken into account (T = 0 K). The magnetization can be reversed only by magnetic fields higher than a critical value, which at 0 K equals the anisotropy field H_k_ and the coercive field H_c_. The hysteresis loop is rectangular, its area is maximum and gives us the upper limit of the SAR for a given material:

(3) $$SAR=P/\rho =Af/\rho =4\mu_{0}M_{s}H_{c}f/\rho \;$$

where A stands for the hysteresis loop area, M_s_ is the saturation magnetization, H_c_ is the coercive field, f is the frequency, and *ρ* is the density. The SAR dependence on the H_max_ is a Heaviside type step function, as no magnetization reversals are produced for the fields below the H_k_.

For randomly oriented MNPs, the H_c_ is lower by 0.48 compared to the H_k_. Consequently, the maximum SAR is reduced in the same field conditions to about 1/2 of the pure Stoner–Wohlfarth NPs [8]. Moreover, considering the possibility of thermal activation, the H_c_ depends on the temperature in a complex manner. By comparing the numerical simulation with this type of formula, a general equation was proposed for calculating the H_c_ and for interpreting the SAR data in the case of F-MNPs [43]:

(4) $$\mu_{0}H_{cHyp}=0.463\mu_{0}H_{k}\{1-{\left[\frac{k_{B}T}{KV}ln\left(\frac{k_{B}T}{4\mu_{0}H_{cHyp}M_{s}Vf\tau_{0}}\right)\right]}^{0.8}\}\;$$

here the H_cHyp_ represents the hyperthermia coercive field, k_B_ is the Boltzmann constant, K is the anisotropy constant, V is the MNP volume and τ_0_ is a characteristic time/frequency factor in the Néel–Brown relaxation times, usually taken in the range 10^−9^–10^−11^ s. This expression applies within a 10% error if the term in the squared brackets denoted κ is smaller than 0.7. In our case, based on the TEM and VSM data, the estimated κ is around 0.25, within the limits of validity of Equation (4). However, Equation (4) does not describe accurately the SAR dependence on the H_max_ for the low field values. This equation is more accurate for the high H_max_ and the shape of this curve describes only the region close to saturation. Recently a slightly changed expression was proposed to describe the SAR dependence on the H_max_ for the immobilized MNPs [44], by adding a sigmoidal type of phenomenological multiplicative factor. In this paper, we use a phenomenological sigmoidal type of logistic function, which we previously have demonstrated [26,27,28,45,46] that provides an excellent fit to the experimental data (R^2^ > 0.999):

(5) $$\mathrm{SAR}={\mathrm{SAR}}_{\max}\frac{{\left(\frac{H}{H_{\mathrm{cHyp}}}\right)}^{n}*\propto }{1+{\left(\frac{H}{H_{\mathrm{cHyp}}}\right)}^{n}*\propto }$$

and

(6) $$\propto =\frac{n+1}{n-1}$$

Whatever equation is used, a sigmoidal dependence of the SAR on H provides three main parameters: (i) the saturation value of the SAR (SAR_max_); (ii) the inflection point of the curve or the hyperthermia coercive field (H_cHyp_), the point of the highest slope in the SAR dependence on the H_max_; _(_iii) the exponent n which shows how steep this dependence, is related in fact to the squareness of the dynamic hysteresis loop. The higher the n, the closer the behavior of the MNPs to the Stoner–,Wohlfarth model. The SAR data measured for the Zn ferrites dispersed in water and PEG 8K presented in Figure 1 were fitted with Equations (5) and (6) and the main parameters derived from the fittings are presented in Table 2.

**Figure 2:**
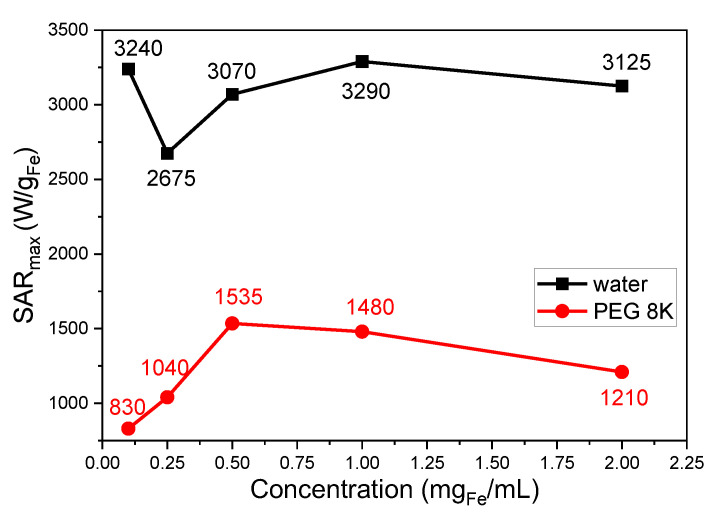
SAR_max_ dependence on the concentration of the Zn ferrites for the samples suspended in water or immobilized in a solid matrix PEG 8K.

As can be seen from Figure 2, the SAR_max_ values vary nonmonotonically with the concentration. As the concentration increases, the SAR decreases to a minimum value for a concentration of 0.25 mg_Fe_/mL, attaining a maximum of 1.00 mg_Fe_/mL. The SAR variation with the concentration was a matter of dispute in the MH literature, as contradictory data were reported by several groups [18,19].

We previously reported that the SAR variation with the concentration could be correctly interpreted only by considering the full SAR dependence on the H_max_ for a large range of H_max_ values, able to saturate the SAR. In this sense, we observed experimentally that for the large Mn and Zn ferrite NPs (80 nm), the SAR_max_ decreases as the concentration increases in the concentration range of 1.00–4.00 mg_metal_/mL [28]. However, at small H_max_ values, below saturation, the situation was reversed [28]. This type of behavior could be explained by the change in the H_cHyp_ with the concentration, as we noticed that the H_cHyp_ significantly decreases with the concentration increase. We assigned this type of behavior to the chain formation in water, under the action of the AMF [28]. As the H_chyp_ is smaller at higher concentrations, the SAR reaches saturation at smaller H_max_ values. This leads to a SAR increase with the increasing concentration in this low field range. At the saturating H_max,_ the increase in the dipolar interactions explains the reverse phenomenon, the SAR decreases when the concentration increases (in this concentration range 1.00–4.00 mg metal/mL). 

The nonmonotonic dependence of the SAR on the concentration was also reported by other groups [47,48,49,50,51], and several theoretical models and simulations were proposed to explain the experimental data. Because the nonmonotonicity of the SAR evolution, as a function of the concentration, depends on the relative values of the interaction energies involved. In Table 3, we estimated the values of the anisotropy, dipolar and Zeeman energies for the relevant situations in our study. 

When two MNPs are in contact, the center-to-center distance (d) between them is equal to the diameter (D) leading to the highest magnetic coupling parameter Γ. The data in Table 3 show that the dipolar interaction energy, when the particles are in contact is higher than the anisotropy energy and corresponds to a Zeeman energy for an external magnetic field of around 30 kA/m. The dipolar energy is strongly dependent on the distance between the MNPs (being inversely proportional to the third power of the distance) and thus it strongly depends on the concentration. The dependence of the distance between the two MNPs on the concentration, is not straightforward. Assuming that the MNPs are uniformly dispersed in the whole volume, one can obtain easily that the d is related to the volume fraction of the MNPs by d = 0.8Dϕ^−1/3^ (see Section S6 for the calculations), which corresponds to d = 9.35D, a distance for which Γ is smaller than one. However, computer simulations of the nearest-neighbor (NN) distances between MNPs of finite size, show that the mean distance between MNPs is much smaller [52,53]. Moreover, these calculations do not take into account the interaction forces between the MNPs which might significantly affect the data. Very recently, Serantes and Baldomir [54] used the magnetic coupling parameter Γ to calculate the threshold of the agglomeration of the MNPs as a function of their K, so taking into account the magnetic interactions (but not the Van de Waals forces or electrostatic interactions). The results show that as K increases, the permanent magnetic moment of the MNPs increases, and therefore the size threshold for the agglomeration is higher for particles with a smaller K (25 nm for K = 8 kJ/m^3^ as compared to 20 nm for K = 15 kJ/m^3^). Moreover, the results indicate that the fraction volume threshold for the agglomeration is 2 × 10^−3^ for K = 8 kJ/m^3^ but this threshold increases with K and if the MNPs are coated with a thick nonmagnetic layer (a very important observation, especially for biomedical applications of MNPs which requires the biocompatibilization produced by coating) [54]. In our case for the maximum concentration used in this study, the volume fraction is 3.2 × 10^−4^, below this threshold of agglomeration for the particles with a diameter of 27 nm and the anisotropy constant in the range of 10^4^ J/m^3^. 

Conde-Leboran et al. [48] explained the nonmonotonicity of the SAR dependence on the concentration by considering the passing of the MNPs from a single-NP regimen to a collective behavior. As such, in the case of F-MNPs, at very low concentrations, the magnetization reversal occurs when the Zeeman energy is large enough to surpass the local energy barrier felt by each MNP, set by the magnetic anisotropy. As the concentration increases, the interactions between F-MNPs lead to an overall decrease in the anisotropy energy barriers and, consequently, a reduction in the SAR. By further increasing the concentration, the mean distances between MNPs decrease, and the energy of the MNP next-neighbour (NN) interaction increases. The system passes from a single-MNP regimen to a collective behavior. The MNPs are submitted to a field composed of the external AMF and the dipolar NN interaction field. The minimum in the SAR_max_ evolution as a function of the concentration occurs at the concentration threshold between the individual MNP and the collective behavior as a result of the competition between the anisotropy energy and the dipolar interaction energy [48]. Above this minimum, the dipolar interaction energy surpasses the anisotropy energy, and in this collective regimen, there is competition between the dipolar field and the applied AMF. The SAR developed in the suspension depends on the relative ratio between the H_max_ and the dipolar field. The H_max_ value should be large enough for a given concentration to overcome the local dipolar field and achieve a significant hysteresis loop. While the H_max_ increases, the SAR increases, until the saturation is reached. As the concentration continues to increase, the field needed to reverse the magnetization increases as well, and therefore, the larger SAR values are reached, provided that the applied AMF is large enough to obtain a major hysteresis loop. However, this mechanism does not function at higher concentrations, due to the transition to minor loops [48]. 

Carrey et al. [50] have simulated randomly-distributed and randomly-oriented MNPs with various diameters, including MNPs in the blocked state. Their paper shows that magnetic interactions due to increasing MNPs concentration, increase both the H_c_ and the saturation field simultaneously and thus the hysteresis loop area. The increased coercivity widens the AC hysteresis loops while the increased saturation field results in a transition to minor hysteresis loops; thus, the hysteresis area (and the SAR) first increases and then decreases with the concentration [50]. However, this simulation shows that for a very large AMF (of about 1T, a field that is not experimentally reachable in MH setups) no maximum occurs, and the SAR increases monotonically with the concentration [51], a theoretical result that is not physically covered. 

Ovejero et al. [49] compared the simulation results to the experimental data obtained on 20 nm iron oxide NPs. The simulation was found to fit the experimental alternating current (AC) hysteresis curves only when the anisotropy axes of the simulated MNPs are aligned. The increase in the SAR with the concentration is attributed to an increase in the magnetization of the ensemble due to the interparticle interactions mediated by the inter-aggregate dipolar interactions. On the other hand, the increase of iron oxide NPs aggregate size leads to a progressive reduction of the SAR values related to the demagnetizing effects mediated by intra-aggregate dipolar interactions. In this case, the H_c_ remains constant throughout [49]. 

Similar results of the SAR dependence on the concentration were reported more recently by Bae et al. [51] with a non-monotonically dependence of the SAR on the concentration in the range of 0.10–40 mg_Fe_/mL. The model for explaining these findings identifies four concentration ranges with different interaction regimens. In the low concentration range (0.10 mg_Fe_/mL) the SAR is maximum due to no or negligible interactions between MNPs, characterized by a high H_c_ and the magnetization, a strong incoherent mode characterizes the magnetization reversal. As the concentration increases (for the concentration in the 0.50 mg_Fe_/mL range) the dipolar interactions increase, leading to a decrease in the H_c_ and the magnetization, and the SAR. A weak coherent mode characterizes the magnetization reversal and the SAR reaches a minimum. As the concentration continues to increase (in the 1–10 mg_Fe_/mL range), the SAR reaches a second maximum. This second maximum proves that the dipolar interaction energy (E_dip_), which increases monotonously with the concentration, is not the only interaction responsible for the changes in the SAR. Other magnetostatic interactions, compete with E_dip_ in this concentration range, such as the magnetic potential energy (E_p_) directly related to the magnetic stray field coupling energy and the uniaxial anisotropy energy, together with the exchange energy (E_ex_) between adjacent MNPs or spins. Although the E_dip_ increase with the increasing concentration, in this range, the E_p_ and the weakly generated E_ex_ are comparable to or larger than the E_dip_. That is why a chain-like incoherent fanning mode of the spins can be formed in the adjacent MNPs. This causes the easy spin rotation of the adjacent MNPs due to a lowered total energy barrier under the AMF with an increase in the magnetization and a faster relaxation caused by the fanning mode of the spin rotation. A further increase in the concentration (above 10 mg_Fe_/mL) can lead to the formation of prolate spheroids or chain-like structures producing large magnetostatic stray fields, demagnetizing adjacent MNPs, thus reducing the magnetization [51].

Our data could be interpreted qualitatively within the frame of the models mentioned above, with slight changes, using the fitting parameters’ (SAR_max_, H_cHyp_, and n) dependence on the concentration. The minimum in the SAR evolution with the concentration (in our case, 0.25 mg_Fe_/mL), when the concentration increases from 0.1 mg_Fe_/mL, it can be explained by the passage from the individual to a collective behavior in the low concentration range. The decrease in the SAR_max_ as the concentration increases, in the very low concentrations range (0.10–0.25 mg_Fe_/mL) can be explained by the decrease in the H_cHyp_ from 23 kA/m to 20 kA/m (Figure S6). This means a decrease in the dynamic hysteresis wideness (i.e., its area) resulting, thus in a SAR drop. As we continue to increase the concentration above 0.25 mg/mL, the H_cHyp_ values are almost constant (around 20 kA/m, Figure S6 left), as it was also obtained in the theoretical modeling [50], and therefore the further changes in the SAR can be attributed only to changes in the dynamic magnetization and the squareness of the dynamic hysteresis loop. The SAR_max_ (reached for a concentration of 1.00 mg/mL) is a result of an increase in the ensemble magnetization due to the interparticle interactions, as previously proven by the theoretical and numerical works [49,50,51]. However, increasing the concentration above 1.00 mg_Fe_/mL, leads to the formation of aggregates with larger sizes which in turn have a demagnetizing effect because of the intra-aggregate dipolar interactions, thus reducing the magnetization of the ensemble, which finally reduces the heating performance of the Zn ferrites. It is worth mentioning that in our recent paper [35], the same Zn ferrite MNPs coated by a silica shell in relatively small clusters, comprising only a few MNPs, do not show any concentration dependence of the SAR in the concentration range 0.25–1.00 mg_Fe_/mL. This means that all of the characteristics are the same (within the experimental errors), including the SAR values, the H_cHyp_, and the exponent n. The clusterization within the small silica-coated structures, leads to the enhancement of the dipolar interactions between the MNPs within a cluster. This phenomenon is the reason for the drop of the H_cHyp_ to 18 kA/m (as compared to 20–23 kA/m for the individual “naked” MNPs, Figure S6 left) and the subsequent SAR_max_ drop to around 2600 W/g_Fe_. The decrease in the SAR of these silica-coated small clusters might be explained in the frame of the above-presented model. At low concentrations, the Zn ferrites are already in a collective regimen within these small clusters, exhibiting a reduced H_c_, which reduces the width of the dynamic hysteresis area and thus the SAR. Increasing the concentration of the aggregates does not influence the SAR as the silica coating is thick enough to maintain a significant distance between the magnetic cores of these clusters, thus keeping the inter-aggregate interaction energy small. The intra-aggregate interaction energy stays constant because the MNPs are fixed in the silica shells and are not dependent on the number of aggregates (clusters) because increasing the number of clusters doesn’t affect the intracluster interactions [35]. 

Coming back to the present study, because above 0.50 mg_Fe_/mL, the H_cHyp_ remains almost constant, the only reasonable explanation for the maximum SAR_max_ at 1.00 mg_Fe_/mL is that, the increase in the concentration between 0.50–1.00 mg_Fe_/mL leads to an increased magnetization of the ensemble. Increasing the concentration, above 1 mgFe/mL, leads to a decrease in the magnetization. This hypothesis is supported by other results from the literature [22], showing that by using both AC hysteresis data and numerical calculations, after the initial decrease the H_cHyp_ remains constant with the increasing concentration. The changes in the SAR and the maximum are explained by an increase in both M_s_ and the remanent magnetization (M_r_) of the ensemble, which increases also the squareness of the loop, and subsequently the SAR. This increase in the squareness of the hysteresis loop at 1.00 mg_Fe_/mL is revealed by the highest value of the exponent n (Figure S6 right) for this concentration. The exponent n, which, as discussed earlier, is directly related to the squareness of the dynamic hysteresis loop (it is related to the M_r_/M_s_ ratio), increases by increasing the concentration from 0.1 mg_Fe_/mL up to 1 mg_Fe_/mL and afterward decreases, thus reaching its maximum value of 5.6, at this latter concentration. Moreover, it was hypothesized that this increase in the magnetization with the increasing concentration might be explained by the tendency of MNPs (especially those with low anisotropy) to organize themselves in chains, the chain structure increasing the anisotropy of the ensemble [22]. 

### 3.2. Magnetic Hyperthermia of Zn Ferrites Immobilized in PEG 8K 

We performed MH experiments with the Zn ferrites randomly dispersed in a solid matrix, namely PEG 8K. We noticed a dramatic decrease in the SAR values, in general by 50%, after the Zn ferrites were immobilized in the solid matrix, as compared to the samples measured in water (Figure 1), the SAR_max_ was around 1500 W/g_Fe_ at a concentration of 0.50 mg_Fe_/mL (Figure 2). This decrease in the SAR with immobilization originates in the inhibition of the Zn ferrites rotation, which blocks the Brown mechanism’s contribution to the absorption power. The measurement of the heating performances of MNPs in media with various viscosities, trying to mimic their reduced mobility in a biological environment, is relevant for biomedical applications. This significant decrease in the SAR for immobilized samples reveals that the Brown mechanism is one of the main mechanisms involved in the heat release and, at first view, would make our MNPs less suitable for biological applications. However, even with a 50% decrease in the SAR, due to immobilization, the Zn ferrites can provide SAR values above 1 kW/g_Fe_ at 355 kHz and H of around 30 kA/m, which, with a proper dosage can lead to efficient heating in most in vitro or in vivo applications, close to the safety conditions [6,7]. 

However, it was also theorized that the immobilization of the MNPs in the solid matrix impedes their organization in the chains under the influence of the AMF [22]. Therefore the effect of the chain organization leading to an increased magnetization cannot be produced in the media with a reduced mobility [22]. Experimental and theoretical results have shown that the effect of chain organization leads to an effective anisotropy (K_eff)_ which will be different from the K of the individual MNPs. The K_eff_ is the value that individual MNPs would have to display a H_c_ similar to the one measured. This effect of the chain organization can be seen only in the case of low anisotropy MNPs when the interaction with the AMF is stronger, as compared to the anisotropy energy, which in our case is valid as the AMF is larger than about 20 kA/m (see Table 3). It was proposed that the K_eff_ could be obtained by fitting the hyperthermia data with an equation similar to Equation (4), by changing the numerical pre-factors, as presented in Section S7 (Equations (S1) and (S2) for the aligned and randomly oriented samples, respectively) [22].

By solving numerically these two equations, we obtained the K_eff_ values decreasing from 8 kJ/m^3^ to 7.1 kJ/m^3^ when the H_cHyp_ decreases from 23 kA/m to around 20 kA/m, for the samples aligned with the field and dispersed in water (using Equation (S1)). For the immobilized samples, we considered the randomly oriented case (Equation (S2)) and we obtained the K values slightly decreasing from 17.6 kJ/m^3^ to 15.2 kJ/m^3^ for a H_cHyp_ decreasing from 29.7 kA/m to 24.2 kA/m. These values were obtained by assuming a M_s_ of 380 kA/m, corresponding to the VSM measurements. Nevertheless, it is important to mention that the saturation magnetizations reached during the AC hysteresis loops are significantly smaller, as compared to the DC-VSM determinations [22] and more appropriate calculations would involve using the M_sat_, the saturation magnetization from the dynamic hysteresis loops instead of the M_s_.

Anyway, using these K_eff_ values, one can observe that σ, the ratio between the anisotropy and thermal energy (Table 3) is reduced to the range of 18–20 for the samples in water and is increased to above 40 for the immobilized samples, as they are larger than the magnetic coupling parameter and equivalent to the Zeeman energy for an external field of around 30 kA/m, very close to the H_cHyp_. Both the AC hysteresis measurements and the numerical simulations have shown that this decrease in the K_eff_ is accompanied by an increase in the remanence ratio (M_r_/M_s_), an increase in the squareness of the AC hysteresis loop, and finally its area, which is directly related to the SAR [22]. It is worth noting that in our analysis, the squareness of the dynamic hysteresis loop is related to the exponent n, which, except for the smallest concentrations, is significantly higher for the samples measured in water (Figure S6 right), which explains the higher SAR values measured in water.

The SAR_max_ increases when the concentration increases from 0.10 to 0.50 mg_Fe_/mL, and, as the concentration is further increased, the SAR decreases (Figure 2). This type of dependence, lacking the decrease of the SAR with the increasing concentration in the diluted samples, could be easily explained by the fact that the Zn ferrites are uniformly dispersed and immobilized within the PEG 8K matrix. The immobilization of the Zn ferrites keeps their position fixed, not allowing their association to reduce the energy barrier needed to reverse their magnetization as in the case of the water suspensions. This explanation is also supported by the much higher values of the H_cHyp_ in the range of 27.35–29.66 kA/m (Figure S6 left) for the immobilized samples. The only increase in the SAR with the concentration up to 0.50 mg_Fe_/mL for a quasi-constant constant H_cHyp,_ can be explained by an increase in the magnetization (the magnetizing effect in this concentration range). At concentrations above 0.50 mg_Fe_/mL, the SAR decreases by a demagnetizing effect and by a slight H_cHyp_ decrease. The mechanism behind the SAR drop is the increase in the dipolar interaction energy at a slightly increased concentration.

### 3.3. Magnetic Hyperthermia of the Zn Ferrites Dispersed in Water under the H_DC_ of 10 kA/m (H_DC_ < H_c_)

As mentioned above, several reports have shown that for SP-MNPs, the SAR increases by almost 40% if a small H_DC_ is parallel to the AMF on the samples dispersed in water [32]. Moreover, the time-resolved MH measurements performed by the dynamic hysteresis [30,31] showed that during MH, the chains are formed under the influence of the AMF, and the chain formation increases both the squareness of the dynamic hysteresis loop and the SAR. Therefore, our goal was to check if an H_DC_ applied both parallel or perpendicular to the lines of the AMF could influence the heating performance of the Zn ferrites dispersed in water. In the first step, we check the effect of high H_DC_ on hyperthermia, and we notice that an H_DC_ around 200 mT can block the Zn ferrites in the field direction and stop their heating in the AMF. Several groups previously reported this observation and it was proposed to be used for finely controlling the spatial localization of MH. By creating the H_DC_ around a tumor and allowing a zero H_DC_ in the tumor area, such as in the case of the magnetic particle imaging technique, we would eventually be able to obtain localized heating by MH only in the tumor area [55].

An H_DC_ of 10 kA/m, below the H_c_ of the Zn ferrites (15 kA/m), has been firstly superposed on the AMF. We checked if such a field can induce the chain formation in the magnetic colloid. The samples containing MNPs were disposed on the TEM grid under a DC magnetic field of 10 kA/m and they were allowed to dry before they were introduced into the microscope. As can be seen in Figure S7, the MNPs dispose themselves mostly in chain-like structures, supporting thus our hypothesis. 

**Figure 3:**
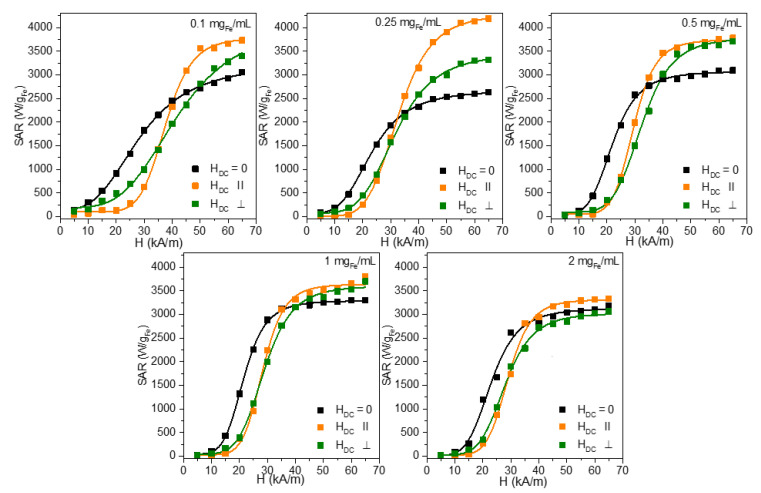
SAR dependence on the H for both the parallel or perpendicular orientations of the H_DC_ (10 kA/m) to the AMF lines for Zn ferrites dispersed in water in the concentration range 0.10–2.00 mg_Fe_/mL. For comparison, the SAR dependence on the H for all samples in zero H_DC_ is also plotted.

The dependence of the SAR on the H_max_ for different concentrations, ranging from 0.10 mg_Fe_/mL to 2.00 mg_Fe_/mL, is presented in Figure 3, while Table 4 summarizes the main parameters derived from the fitting with the Equations (5) and (6). Both orientations of the H_DC_ can significantly increase the SAR_max_, except for the perpendicular configuration at 2.00 mg_Fe_/mL. However, a higher enhancement has been recorded for the parallel configuration (Figure S8a). The highest enhancement of 60% for the SAR_max_ was obtained for a concentration of 0.25 mg_Fe_/mL for the parallel orientation, reaching a very high value of 4.3 kW/mg_Fe_. It is pretty interesting to notice that at this concentration, the SAR_max_ reaches a minimum value without the H_DC_ and the maximum value with the H_DC_. This concentration marks the limit for passing from a single MNP behavior to a collective one in the absence of an H_DC_. The occurrence of the H_DC_ field enhances the probability of the chain organization of the MNPs, parallel to the AMF, and the increase in the magnetization might be noticed even at lower concentrations. The H_cHyp_ values are shifted in the presence of an H_DC_ field with 8–10 kA/m for all concentrations except 0.10 mg_Fe_/mL, where the shift is significantly higher for both field orientations (13 kA/m, Figure S8b). This shift is explained by the interaction of the Zn ferrites with the H_DC_ which locks the Zn ferrites parallel with the H_DC_ lines. Only when the H_max_ surpasses the H_DC_, the Zn ferrites change their orientation and the effective heat release is recorded only above 15 kA/m. However, the H_cHyp_ concentration dependence is qualitatively similar for all three H_DC_ conditions (zero, parallel, and perpendicular), with a steep decrease in the lower concentration range, followed by an almost constant value (Figure S8b).

The exponent n from Equations (5) and (6), related to the squareness of the dynamic hysteresis loop, is increased in the presence of the H_DC_ to significantly much higher values (5.6–8), in the case of the parallel configuration (Figure S8c). This behavior is translated into a much steeper dependence of the SAR on the H, as can be seen in Figure 3. Interestingly, in the case of the parallel configuration, the minimum n of 5.6 occurs at 0.25 mg_Fe_/mL, for which the SAR_max_ is the highest. The SAR_max_ values are proportional to the area of the dynamic hysteresis loop and increase with the increase in the H_cHyp_, exponent n (squareness), and magnetization. The highest SAR_max_ obtained at 0.25 mg_Fe_/mL might be explained in these conditions only by a significant increase in the sample’s magnetization. We presume that the effect of the H_DC_ is to increase the probability of the association between the individual Zn ferrites. This probability is also a function of the concentration because the mean distance between the Zn ferrites decreases with the increasing concentration. Under the H_DC_ field, the Zn ferrites align themselves along the lines of the H_DC_ increasing thus the mutual attraction. We believe that this interaction favors the formation of small chain-like structures containing only a few Zn ferrites. Niculaes et al. [56] have shown that the association of MNPs in dimers and trimers, i.e., in structures containing a small number of MNPs, can significantly enhance their heating properties, while their organization in centrosymmetric structures comprising more MNPs lowers their SAR values. As the concentration increases, the H_DC_ field still can organize the MNPs in chain-like structures; however, these structures will deviate from a one-dimension (1D) organization. As the chain is composed of a larger number of MNPs, thermal fluctuations also lead to a partial misalignment of the MNP magnetic moments from the direction of the field, the increase in the SAR being less pronounced. As shown by the experiments performed on MNPs aligned under an H_DC_ either on phantom gels or in vitro in cells loaded with MNPs, the SAR of the structure depends on the quality factor Q, a parameter reflecting the similarity of the structure to a 1D one [21]. The dipolar field (H_D_) sensed by a particle in the chain can be expressed as:

(7) $$\vec{H_{D}}=M_{S}{\left(\frac{D}{d}\right)}^{3}Q\vec{f}$$

where D is the diameter of the nanoparticle, d is the mean distance between two neighboring nanoparticles, Q is the quality factor and$\vec{f}$ is a vector related to the geometry and orientation of the chain/cylinder to the external magnetic field [23].

As the structure is closer to a 1D one, the Q increases, and consequently the SAR. Therefore, we can explain qualitatively our data based on this model considering that at a concentration of 0.25 mg_Fe_/mL, the Q is the highest, and the structure produces the highest SAR. As the concentration increases, the Q decreases as the structure evolves toward a more cylindrical geometry, leading to a decrease in the SAR. 

The chain organization of MNPs and how this organization can increase the SAR were systematically investigated in magnetosomes [57]. The hyperthermia properties of magnetosome chains could be described theoretically by the Stoner–Wohlfarth-based model (SWBM) within the frame of the high energy barrier approximation [8,57], by assuming that thermal energy, k_B_T, is much smaller than the energy barrier between the two minimum energy states K_eff_V, where K_eff_ is the effective anisotropy energy density and V, the MNP volume. In essence, this model assumes noninteracting MNPs. The effect of the chain organization of the individual magnetosomes could be integrated into the model only considering a biaxial anisotropy [58]. Apart from the intrinsic MNPs anisotropy, the influence of the other MNPs within a chain is introduced as a second anisotropy constant called extrinsic anisotropy, which is oriented along the direction of the chain [50]. The energy density can be written as:

(8) $$E\left(\theta ,\varphi ,t\right)=K_{1}\left[1-{\left(\vec{u_{1}}\;.\vec{u_{m}}\;\right)}^{2}\right]+K_{2}\left[1-{\left(\vec{u_{2}}\;.\vec{u_{m}}\;\right)}^{2}\right]-{\;\mu }_{0}\mathrm{MH}\left(\vec{u_{H}}.\vec{u_{m}}\right)$$

where u_1_ is the unit vector of the easy axis, u_2_ is the unit vector of the extrinsic anisotropy (chain direction) and u_m_ and u_H_ are the unit vectors in the direction of the magnetization and the external field, respectively. If K_2_ = 0, we have the case of an isolated noninteracting MNP, which is solved according to the model proposed by Carrey et al. [8]. Based on Equation (8), it was shown that the energy landscape presents two minima, one in the positive direction and the other in the negative direction of the z axis, and it can be calculated numerically by using the double-well model. The model which considers the biaxial anisotropy correctly describes the SAR dependence on the H_max_ of the chain-like structured MNPs. In contrast, either the cubic anisotropy or uniaxial anisotropy, including the effect of the polydispersivity of MNPs, cannot correctly describe the experimental data [57].

The SAR enhancement of the SP-MNPs under an H_DC_ was earlier reported [32,58] and was also explained by the increase in the anisotropy energy due to the chain formation. Optical microscopy and atomic force microscopy (AFM) indicated the chain formation under an H_DC_ of 80 G, with a larger and thicker chain with the increasing concentration [58]. The energy landscape of an MNP within a chain was proposed to be described by the following equation [18]:

(9) $$E=-\left[\frac{3V^{2}M_{S}^{3}}{{\left(D+d\right)}^{3}}\sum_{i=1}^{Q}\frac{Q-i}{{\mathrm{Qi}}^{3}}+K_{\mathrm{eff}}V\right]{\sin\theta }^{2}-\mu_{0}M_{s}{\mathrm{VH}}_{\mathrm{DC}}\cos\left(\theta -\varphi \right)$$

where Q represents the number of particles within a chain, D is the diameter of the nanoparticles, and d is the distance between two MNPs (surface to surface). The first term in Equation (9) represents the dipole-dipole interaction and the second term indicates the effect of the chain formation, which can be translated into an increase in the K_eff_ along the direction of the H_DC_. For the case Q = 1 (a single MNPs) Equation (9) gives the well-known Stoner–Wohlfarth energy. Based on this equation, the energy presents two minima with an anisotropy energy barrier increasing with the Q. This indicates an increase in the K_eff_ along the direction of the H_DC_, the increase being significant for a few MNPs within the chain with saturation for the chain containing a higher number of MNPs. This model could explain the increase in the SAR of the suspensions of the MNPs submitted to an H_DC_ but cannot explain the decrease in the SAR if the number of MNPs within a chain surpasses a certain threshold. As one can easily observe, the main difference between Equations (8) and (9) is that in the first case, the chain direction is taken along the anisotropy axis, a hypothesis that is an oversimplification.

Summarizing these theoretical models, we could conclude that, as a standard feature, all of these models show that the effect of the H_DC_ field is to create chains which lead to an increased anisotropy and subsequently the SAR. The MNPs will form chains along the lines of the magnetic field and will tend to orient their easy axis along the field direction to minimize the Zeeman energy. This alignment leads to a steeper transition near the coercive field. In a 1D chain, the magnetization reversal is produced by the reversal of the magnetization of each MNP and the reversal of the magnetization of a particle within the chain will trigger the reversal of the magnetization of the other MNPs along the chain, due to the dipolar interaction. This propagation of the reversal along the chain will narrow the field values around which it occurs (as compared to the randomly distributed samples), increasing the magnetization remanence (i.e., the squareness of the dynamic hysteresis loop). However, as the concentration increases, the structures formed deviate from the 1D chain, which in turn can decrease the anisotropy and reduce the SAR, as observed in our experimental data. 

Surprisingly, when the H_DC_ is applied perpendicular to the direction of the AMF, a significant increase in the SAR is obtained. In this case, the SAR_max_ landscape, as a function of the concentration, is similar to the one recorded in the absence of the H_DC_ (except for the 2.00 mg_Fe_/mL). It exhibits a shift upwards in all SAR values in the presence of the H_DC_ of about 1000 W/g_Fe_ (Figure S8a). 

Our result contrasts with other experimental and theoretical approaches in which, for other types of MNPs, it was shown that an H_DC_, perpendicular to the AMF, decreases the SAR [33,59]. However, micromagnetic simulations have shown that a 20 Oe static field applied perpendicular to the oscillating field, approximately doubles the energy loss [60]. Moreover, a perpendicular static field can increase the effectiveness of a sinusoidal waveform without bringing benefits to the effectiveness of a square waveform [61].

However, other experimental results have shown that MNPs partially immobilized in the agar of different concentrations, can be used to produce mesoscopic chain-like structures with increased heating performances (2 kW/g_Fe_) with heating properties not very sensitive to the direction of the chain to the AC field, in low viscous media [21]. In this sense, the experiments performed with the magnetite MNPs of 44 nm, suspended in hot agar solutions, in the agar concentration range of 0.10–2.00%, and submitted to an H_DC_ during the cooling process, revealed that indeed the parallel orientation produced the highest increase in the SAR (in respect to the random orientation) for the entire agar concentration ranges, but also the 90° orientation increases significantly the SAR up to 0.5% of the agar concentration. In contrast, for the 45°, the SAR increased as compared to the control by 1% [21]. Our results seem to agree with this report and show that the structuration produced by the H_DC_ can effectively increase the SAR. It is unclear if the chains formed generally to the direction of the AMF lines are preserved, or if the combined effect of the H_DC_ and H produces small structures with better heating properties, compared to the case when only the AMF is applied to the suspension. However, this increase in the SAR for the perpendicular configuration of the H_DC,_ is valid only for small concentrations. At 2.00 mg_Fe_/mL, the AMF alone is more effective for heating.

It is also worth mentioning that the combination of the AMF with a perpendicular H_DC_ produces a rotating field, which changes its orientation between +/− arctang(H_DC_/H_max_) at the frequency of the AMF and with a magnitude varying between H_DC_ and (H_DC_^2^ + H_max_^2^)^1/2^. Low-frequency rotating magnetic fields with elongated or quasispherical MNPs, were proposed in recent years for mechanically destroying cancer cells [62,63]. However, we believe that further studies are needed to determine the heat produced by the MNPs in the rotating magnetic fields. Another point to consider is that the effect of the H_DC_ superposed to the AMF one, is to increase the amplitude of the resultant field, which can reduce both Brown and Néel relaxation times. For many MNPs, the Brown relaxation time is shorter under the usual MH conditions, and reducing the Néel relaxation time would be a possible way to increase the SAR for immobilized MNPs.

### 3.4. Magnetic Hyperthermia of the Zn Ferrites Dispersed in Water under H_DC_ of 20 kA/m (H_DC_ > H_c_)

**Figure 4:**
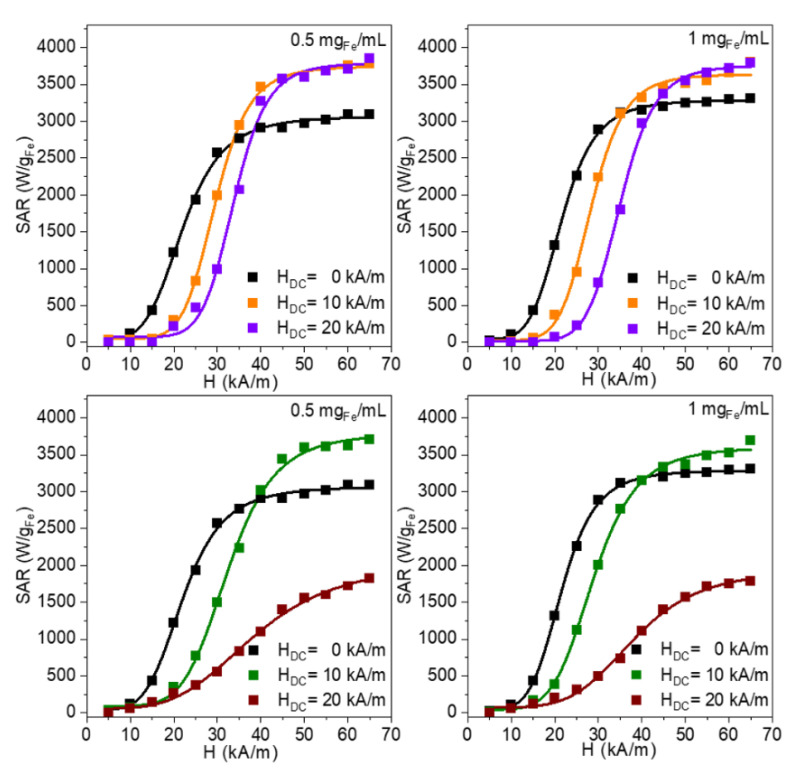
SAR dependence on the H for both parallel (upper panels) or perpendicular (lower panels) orientations of the H_DC_ (20 kA/m) to the AMF lines for the Zn ferrites dispersed in water in the concentration of 0.50 and 1.00 mg_Fe_/mL. For comparison, the SAR dependence on the H for all samples, in zero H_DC_ and H_DC_ of 10 kA/m, is also plotted.

The second set of MH experiments was performed with both parallel and perpendicular configurations, by using an H_DC_ of 20 kA/m, which is higher than the H_c_ (15 kA/m) (Figure 4) for two concentrations (0.50–1.00 mg_Fe_/mL), the main fitting parameters are presented in Table S1.

In the case of the parallel configuration, no significant increase in the SAR_max_ could be detected, when the H_DC_ was increased from 10 kA/m to 20 kA/m (Figure 4). However, there is a clear shift in the curves toward the higher H_max_, as the H_DC_ is increased at 20 kA/m, the H_cHyp_ increases to 33.8–35 kA/m. Moreover, we observed an increase in the squareness of the dynamic hysteresis loops manifested by an increase in the exponent n from 7.5–7.6 to values of 8.8–8.9 (Table S1). For the perpendicular configuration, instead, the SAR decreases dramatically with the increasing H_DC_ from 10 kA/m to 20 kA/m. Furthermore, the H_cHyp_ values increased towards 39 kA/m, significantly larger than in the case of the parallel configuration, while a significant drop in the values of the exponent n is recorded (Table S1). It is quite obvious that, for the parallel configuration, the effect of the H_DC_ on the SAR saturates, with the cost of increasing the H_cHyp_ and the H_max_ needed to reach saturation. In the case of the perpendicular configuration, the H_DC_ increase is detrimental to the heating performances of the MNPs. An H_DC_ of 20 kA/m leads to the formation of longer and thicker chains perpendicular to the AMF lines, within which a demagnetizing effect might occur due to the strong dipolar interactions among the Zn ferrites. Therefore, the magnetization of the assembly is reduced, and consequently, the SAR values drop significantly, compared to the two other cases.

### 3.5. Magnetic Hyperthermia of the Zn Ferrites Immobilized in PEG 8K under H_DC_ of 10 kA/m

**Figure 5:**
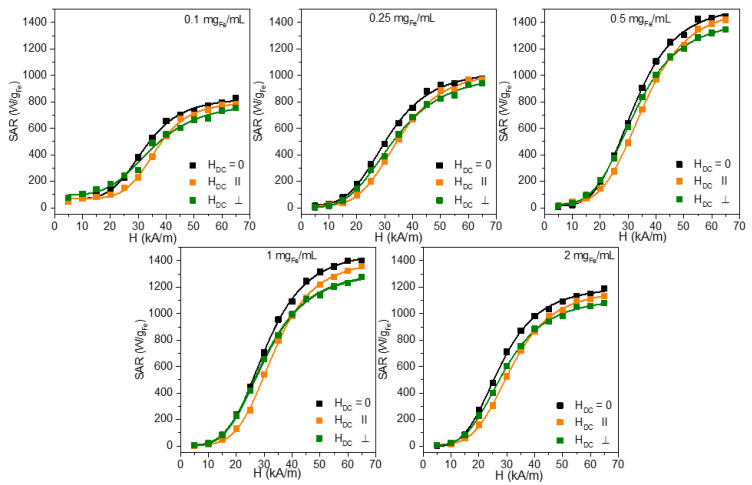
SAR dependence on the H for the Zn ferrites immobilized randomly in PEG 8K and submitted after cooling to the combination of the AMF and H_DC_ (10 kA/m) in the parallel and perpendicular configurations in the concentration range of 0.10–2.00 mg_Fe_/mL.

We also investigated the heat released by the Zn ferrites when they were first randomly frozen in PEG 8K, which is solid at the temperatures reached during MH, and afterward, they were submitted to the combination of the AMF and the H_DC_ of 10 kA/m. The SAR dependences on the H_max_ are presented in Figure 5 and the fitting parameters obtained by using Equations (5) and (6) are provided in Table S2.

No significant change in the SAR was noticed when the H_DC_ was applied in the parallel and the perpendicular configurations (Figure S9a). This result is a clear indication that the SAR_max_ raises, recorded in water under the influence of the H_DC,_ are mainly due to how the Zn ferrites organize themselves under the action of the combination of magnetic fields, provided that their environment allows their movement under the action of the fields. However, the SAR_max_ values are larger for the situation without an external H_DC_ field, but within the measurements, errors are the same with the H_DC_ applied in the parallel configuration. For the perpendicular configuration, the SAR_max_ values are slightly smaller. It is interesting also to note that the H_cHyp_ which, in water are upward shifted for both the parallel and perpendicular configurations, in the case of the samples immobilized in PEG 8K, they are shifted only for the case in which the H_DC_ is parallel to the AMF field (Figure S9b). For the perpendicular configuration, the H_cHyp_ is similar to those without the H_DC_ (Figure S9b). The shift is much smaller (3–4 kA/m), less than half as compared to the water dispersion of the MNPs, and the value of the H_DC_ and could be explained by the fact that the magnetic moments of the MNPs are randomly distributed in all directions in space for the immobilized samples. The fact that the H_cHyp_ values are larger only for the parallel configuration is probably due to the spatial configuration of the total magnetic field with respect to the perpendicular configuration. In the case of the parallel configuration, during the half-period for which the AMF and DC fields have the same sense, the MNPs feel a maximum field of H_max_+H_DC_, in the next half-period, when the two fields have opposite senses, the MNP will feel an external field in the new direction only after the instant value of the AMF field surpasses the DC field amplitude. The maximum field amplitude felt in this new direction is H_max_–H_DC_. Moreover, in the case of the perpendicular configuration, as we mentioned above, the total magnetic field executes partial rotations between +/− arctang(H/H_DC_), meaning that at every moment in time, the MNPs will feel a larger magnetic field as compared to the parallel orientation, the change in the magnetization depends on the orientation of the easy axis in respect to the rotating resultant magnetic field. These differences in the H_chyp_ between the parallel and perpendicular configurations produce an interesting situation with the SAR values larger for the perpendicular configuration, as compared to the parallel one for the H_max_ smaller than the coercive field. As H_max_ is increased over the H_cHyp,_ the situation is reversed. We consider that this is indeed a clear example showing that the heating performances of the MNPs should be assessed and correctly interpreted only if the measurements are performed over the entire range of the H_max_, until saturation is reached.

We also checked for these MNPs, if the alignment of the MNPs before their immobilization affects the SAR (Figure S10a and Table S3). The results show that if the samples were pre-aligned parallel with the AMF field, there is a significant SAR rise after the immobilization of the samples, as we noticed in our previous papers [28,42]. Several other studies pointed out this effect of increasing the SAR by the pre-alignment of the MNPs before being immobilized (gelled) [18,23,24,25]. While in most of the studies, the decrease in the SAR for the immobilized samples was attributed to the blocking of the physical rotation of the MNPs (Brown mechanism), in light of the dynamic hysteresis analysis, it was emphasized that for F-MNPs all of the heating properties are derived from the dynamic hysteresis and no specific contribution (e.g., the Brownian one) could be separated from another contribution [22]. Moreover, the larger SAR values in water, as compared to immobilized samples are due to the chain formation in the mobile phase and the creation of an anisotropy axis along the direction of the chain [22]. We would expect the pre-alignment of the MNPs before the immobilization to restore the SAR measured in water. This is not the case, at least for our MNPs, for which we notice a significant increase in the SAR (40%) but not enough to gain back the values measured in water. These differences could eventually be explained by the changes in the relaxation times upon the immobilization, with the passage from a Brown-dominated relaxation mechanism for the samples dispersed in water, to the Néel dominated relaxation time for the immobilized samples. Nevertheless, the field dependencies of the relaxation times should be taken into account when the applied fields approach or surpass the critical field 42-. The SAR drop when the H_DC_ is applied perpendicular to the direction of the AMF field is following the results of Serantes et al. [21] for the magnetite NPs in high agar concentrations.

We also tested the effects of the H_DC_ on the SAR performances of MNPs pre-aligned parallel to the AFM lines and subsequently immobilized in PEG 8K (Figure S10b). We noticed that both orientations of the H_DC_ slightly reduce the SAR_max_ (Table S3). Once again, we observed that in the case of the parallel orientation, the H_cHyp_ is shifted toward the higher fields while in the perpendicular orientation, the H_cHyp_ is the same as in water (Table S3). This effect is translated into a higher SAR for the perpendicular configuration up to the H_max_ of 40kA/m. For example, for a H_max_ of 20 kA/m, the SAR is 300 W/g_Fe_ for the DC with a parallel configuration and is more than double 620 W/g_Fe_ for the perpendicular configuration. This effect is probably due to the differences in the orientations of the total field in the two cases, as explained for the immobilized samples. This observation might have practical applications as both in vivo and in vitro, the recent experimental data have shown that the pre-alignment of MNPs during the cellular uptake [25] or in a bone cement, [64] significantly enhances the MH efficiency when the AMF was applied along the direction of the alignment. Our results suggest that the superposition of a perpendicular static DC field during MH might significantly increase the heat released, as compared to the parallel configuration. 

## 4. Conclusions

We analyzed, in this work, the MH properties of F-MNPs using an analytical phenomenological function, which provided us three main parameters: the SAR_max,_ the hyperthermia coercive field H_cHyp_, and n an exponent related to the squareness of the AC hysteresis loop. 

Our results measured on Zn ferrite MNPs show a nonmonotonic dependence of the SAR on the concentration, with a maximum at very small concentrations (c < 0.1 mg_Fe/_mL) followed by a minimum at 0.25 mg_Fe_/mL, and a second maximum at around 1 mg_Fe_/mL, in agreement with other reports in the literature. The maximum SAR measured at very low concentrations is not relevant for the MH biomedical applications, since the heat generated is not enough to bring the tissue to therapeutical temperatures. This maximum is explained by a single particle behavior, in which the energy barrier is set by the magnetic anisotropy of the MNPs. As the concentration increases the dipolar interactions among the MNPs become relevant, reducing thus the energy barrier, the H_cHyp,_ and thus the SAR. Further increase in the concentration increases the dipolar interactions, the MNPs passing into a collective regime. The increase in the SAR can be explained by an increase in the magnetization as the coercive field remains constant. The concentration corresponding to the maximum value of the SAR sets the threshold for which the effect of the dipolar interactions passes from a magnetizing to a demagnetizing one. Increasing the concentration above this threshold the SAR decreases due to the increase in the demagnetizing effect which is enhanced by the possible agglomeration of the MNPs.

The MH experiments performed by superposing a static 10 kA/m bias DC field on the AMF revealed an increase in the SAR for both orientations of the DC field, parallel and perpendicular to the AMF. While the enhancement effect produced by a parallel DC field was already proved experimentally for S-MNPs, in this study, we show that a similar enhancement in the SAR could be obtained also for the F-MNPs and is explained by the chain organization of the MNPs stimulated by the DC field. Moreover, we show for the first time experimentally, that a perpendicular DC magnetic field can also increase the SAR but only up to a concentration of 1 mg_Fe_/mL. It seems that the structuration induced by the perpendicular DC field could be translated into an improved heating performance of the MNPs. We also envisaged that when the DC field is normal to the AMF field lines, the overall field is increased and executes a partial rotation. Increasing the DC field strength to 20 kA/m produces a significant decrease in the SAR for the perpendicular configuration, while for the parallel configuration of the SAR increase is the same as for the 10 kA/m DC field, as the effect saturates. We strongly believe that more theoretical and experimental studies could optimize the effects of the DC fields and the MH efficiency.

## Figures and Tables

**Table 1:** Magnetic parameters of the Zn ferrites obtained from DC magnetometry *.

Temperature (K)	M_s_ (emu/g)	M_r_/M_s_	H_c_ (kA/m)
4	100	0.32	23
300	72	0.28	15

* magnetic parameters are from ref. [35].

**Table 2:** SAR_max_, H_cHyp,_ and n for the samples measured in water and PEG 8K.

Concentration (mg_Fe_/mL)	Water			PEG 8K		
	SAR_max_ (W/g_Fe_)	H_cHyp_ (kA/m)	n	SAR_max_ (W/g_Fe_)	H_cHyp_ (kA/m)	n
0.10	3240	23	3.1	830	29.66	4.9
0.25	2675	20.07	3.7	1040	27.35	4
0.50	3070	20.24	4.8	1535	28.89	4.3
1.00	3290	20.24	5.6	1480	27.11	4.1
2.00	3125	21.15	4.9	1210	24.17	3.9

**Table 3:** Interaction energies normalized to the thermal energy.

Energy	Formula	Value	Parameter
Dipolar energy ^1^	Γ = μ_0_(M_s_V)^2^/2πd^3^k_B_T	34.8	d = D = 27 nm
		4.4	d = 2D = 54 nm
Anisotropy energy ^2^	**σ** = KV/k_B_T(K = 25k_B_T_B_/V)	25	K = 25k_B_T_B_/V = 10^4^ kJm^−3^
Zeeman energy ^3^	**ξ** = μ_0_VM_s_H/k_B_T	11.9	H = 10 kA/m
		23.8	H = 20 kA/m
		77.3	H = 65 kA/m

^1^ Γ magnetic coupling parameter is defined as the ratio between the maximum of the magnetic dipole-dipole attraction energy and the thermal energy [52]. Ms is the saturation magnetization, V is the volume of the MNPs, k_B_ is the Boltzman constant, T is the absolute temperature (300 K) and d is the distance between the MNPs (center to center), D is the diameter of the MNPs. ^2^ The MNPs are blocked at the RT, the K, anisotropy constant was calculated considering the blocking temperature as the RT, therefore the calculated value (10 kJ/m^3^) is the lower limit for K. ^3^ The Zeeman energy was calculated for three different values of the magnetic field strength H.

**Table 4:** SAR_max_, H_cHyp,_ and n for the samples measured in water under H_DC_ of 10 kA/m.

Concentration (mg_Fe_/mL)	H_DC_ Parallel			H_DC_ Perpendicular		
	SAR_max_ (W/g_Fe_)	H_cHyp_ (kA/m)	n	SAR_max_ (W/g_Fe_)	H_cHyp_ (kA/m)	n
0.10	3785	36.4	8.0	4070	36.5	3.9
0.25	4285	30.7	5.6	3450	28.9	4.6
0.50	3740	28.5	7.6	3785	30.7	6.0
1.00	3640	27.4	7.5	3600	27.2	5.8
2.00	3315	28.0	7.3	3025	26.4	5.7

## Data Availability

Not applicable.

## Supplementary Materials

The following supporting information can be downloaded at: https://www.mdpi.com/article/10.3390/nano12203578/s1, Figure S1: Physicochemical characterization of Zn ferrites; Figure S2: Heating curves for the samples dispersed in water with or without a static DC field applied in a parallel or perpendicular configuration; Figure S3: Heating curves for the samples dispersed randomly in PEG 8K with or without a static DC field applied in a parallel or perpendicular configuration; Figure S4: Pre-alignment of the Zn ferrite NPs in liquid PEG 8K; Figure S5: H_DC_ configurations superposed on the AMF; Figure S6: Fitting parameters of the MH data acquired in water and PEG 8K; Figure S7: TEM images of the samples aligned in a 10 kA/m DC field; Figure S8: Fitting parameters for the MH data acquired in water with and without the H_DC_ of 10 kA/m; Figure S9: Fitting parameters for the MH data acquired on samples randomly immobilized in PEG 8K; Figure S10: Influence of the pre-alignment of the MNPs before the immobilization on the SAR and the effects of the H_DC_ on pre-aligned samples; Table S1: SAR_max_, H_cHyp,_ and n parameters for the MH data under an H_DC_ of 20 kA/m; Table S2: SAR_max_, H_cHyp,_ and n for the MH data for samples randomly immobilized in PEG 8K and submitted to the AMF and an H_DC_ of 10 kA/m both in the parallel and perpendicular configurations; Table S3: SAR_max_, H_cHyp,_ and n parameters for MH data from Figure S9.
