# Supplementary material for: Enhanced Magnetic Hyperthermia Performance of Zinc Ferrite Nanoparticles under a Parallel and a Transverse Bias DC Magnetic Field
Source: Nanomaterials (Basel). 2022 Oct 12;12(20):3578. doi: 10.3390/nano12203578 (PMC9611223; doi:10.3390/nano12203578)
Supplement: Supplementary file 1 [file nanomaterials-12-03578-s001.zip › nanomaterials-1931577-supplementary.pdf]

Supplementary Materials

Enhanced magnetic hyperthermia performance of zinc ferrites nanoparticles under a parallel and a transverse bias DC magnetic field

Constantin Mihai Lucaciu[1*], Stefan Nitica[1], Ionel Fizesan[2], Lorena Filip[3], Liviu Bilteanu[4,5] and Cristian Iacovita[1*]

[1] Department of Pharmaceutical Physics-Biophysics, Faculty of Pharmacy, “Iuliu Hatieganu” University of Medicine and Pharmacy, 6 Pasteur St., 400349 Cluj-Napoca, Romania

[2] Department of Toxicology, Faculty of Pharmacy, “Iuliu Hațieganu” University of Medicine and Pharmacy, 6A Pasteur St., 400349 Cluj-Napoca, Romania

[3] Department of Bromatology, Hygiene, Nutrition, "Iuliu Haţieganu" University of Medicine and Pharmacy, 6 Pasteur St., 400349 Cluj-Napoca, Romania

[4] University of Agronomic Sciences and Veterinary Medicine of Bucharest, Faculty of Veterinary Medicine, Department Preclinical Sciences, 105 Splaiul Independentei, 050097, Bucharest, Romania

[5] Molecular Nanotechnology Laboratory, National Institute for Research and Development in Microtechnologies, 126A Erou Iancu Nicolae St., 077190, Bucharest, Romania

* Correspondence: clucaciu@umfcluj.ro (C.M.L.);cristian.iacovita@umfcluj.ro (C.I.); Tel.: +0040-744-647-854 (C.M.L.); +0040-743-421-247 (C.I.)

S1. Physicochemical characterization of Zn ferrites:

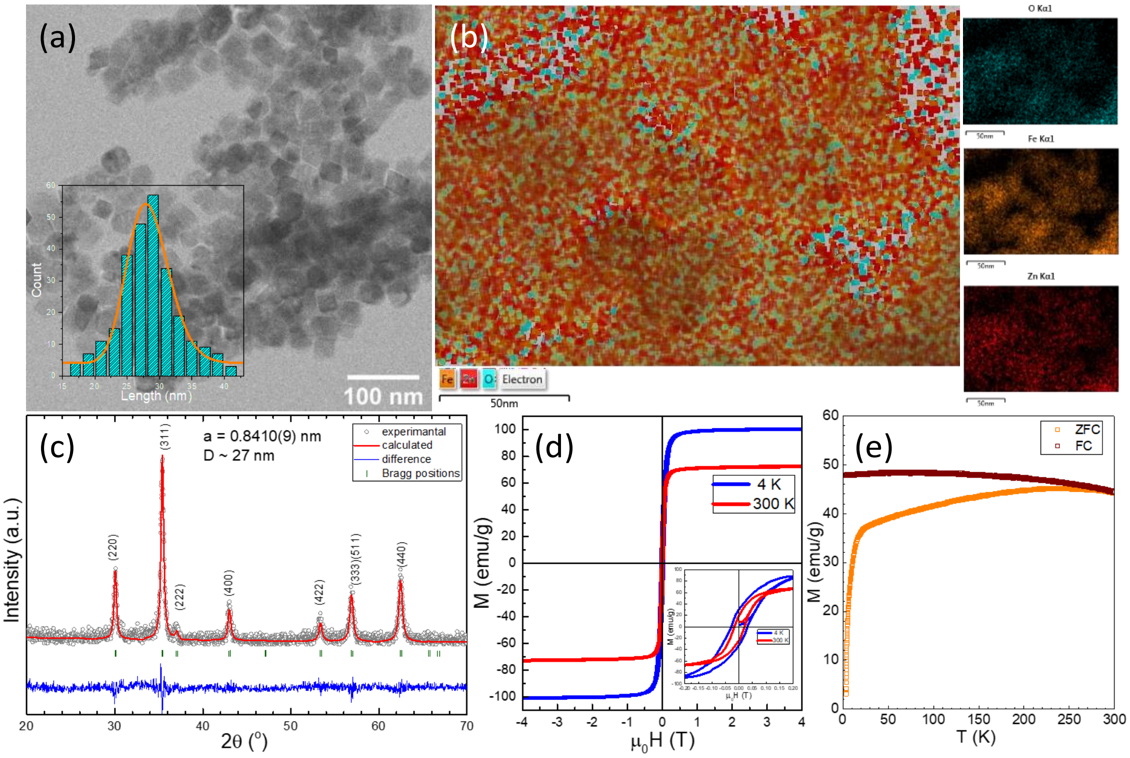

Figure S1. (**a**) TEM images of Zn ferrites NPs. Inset represents the size distribution histogram of Zn ferrites NPs fitted to a log-normal distribution (orange line). (**b**) EDX global chemical map together with chemical maps of Fe, Zn, and O elements of Zn ferrites NPs. (**c**) XRD diffraction pattern of Zn ferrites NPs. (**d**) Hysteresis loops of Zn ferrites NPs acquired at 4 K and 300 K. Inset represents the low-field regime of hysteresis loops. (**e**) Zero field cooled and field cooled magnetization curves of Zn ferrites NPs acquired in an external magnetic field of 50 mT.

S2. Heating curves for the samples dispersed in water with and without the static DC field

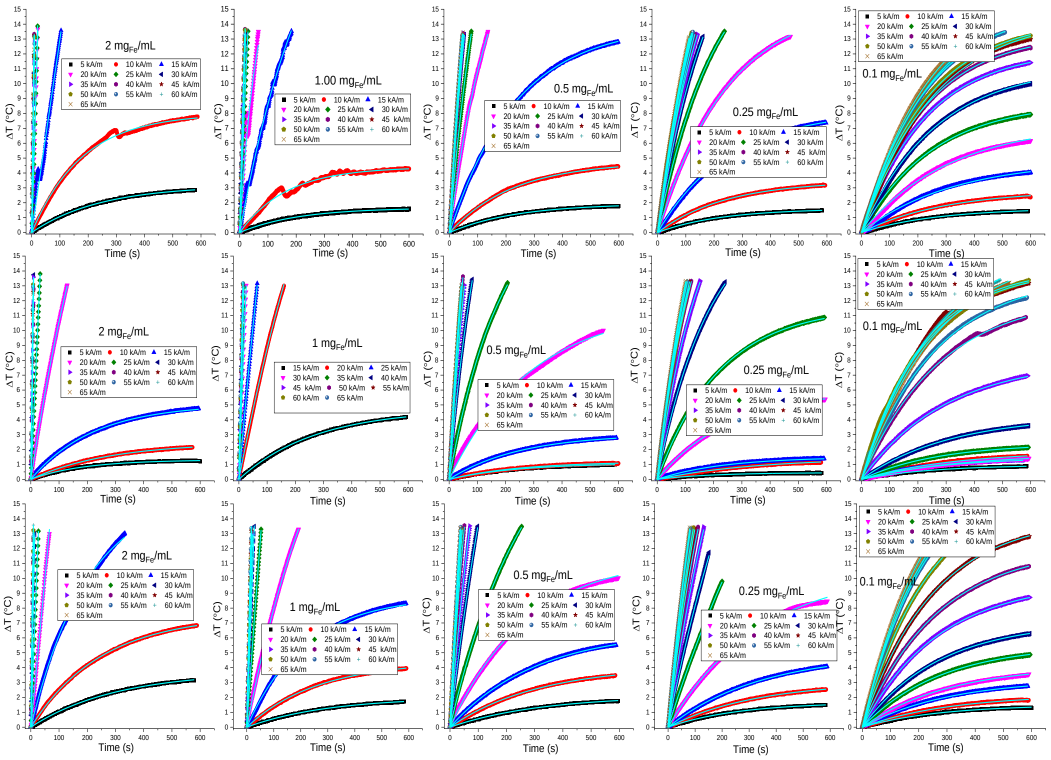

Figure S2 Groups of panels displaying the temperature change ΔT versus time curves fitted with Box-Lucas equation (blue curves) of $Zn_{0.4}Fe_{2.6}O_4$ NPs, dispersed in water without an applied H_{DC} (upper panels) and with an H_{DC} of 10 kA/m applie(midle panels) and perpendicular (lower panels), at an iron concentration ranging from 2.00 mgFe/mL to 0.1 mgFe/mL, recorded as a function of H (5 – 65 kA/m) at a frequency of 355 kHz.

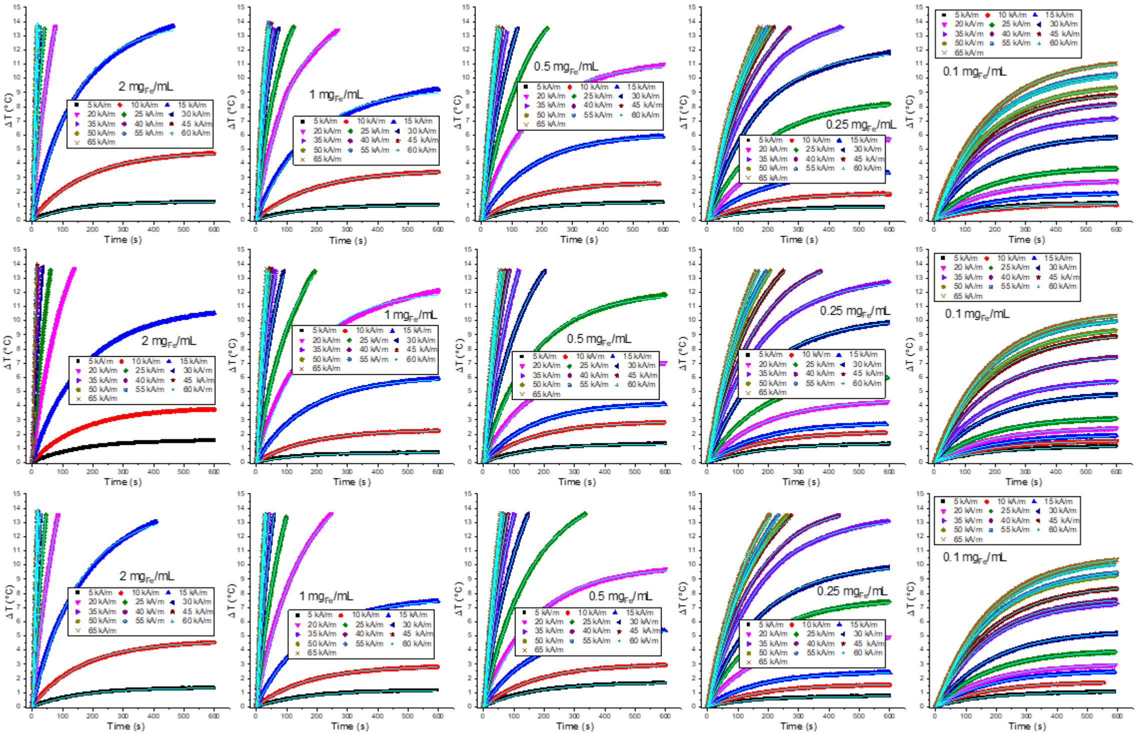

Figure S3 Groups of panels displaying the temperature change ΔT versus time curves fitted with Box-Lucas equation (blue curves) of Zn0.4Fe2.6O4 NPs, dispersed in PEG 8K without an applied HDC (upper panels) and with an HDC of 10 kA/m applied parallel (midle panels) and perpendicular (lower panels), at an iron concentration ranging from 2.00 mgFe/mL to 0.1 mgFe/mL, recorded as a function of H (5 – 65 kA/m) at a frequency of 355 kHz.

S3. Pre-alignment of the Zn ferrites NPs in liquid PEG 8K:

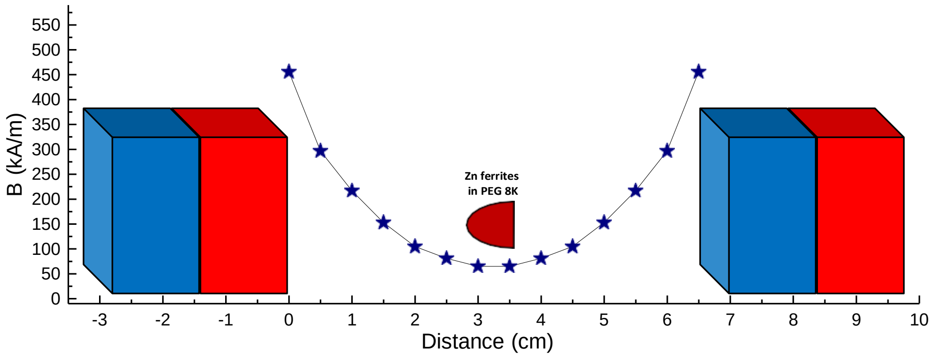

Figure S4. Schematic representation of setup used for the pre-alignment of the Zn ferrites in liquid PEG 8K at 80^{0}C under a magnetic field of 65 kA/m, maximum attainable in MH experiments.

S4. H_{DC} configurations superposed on the AMF:

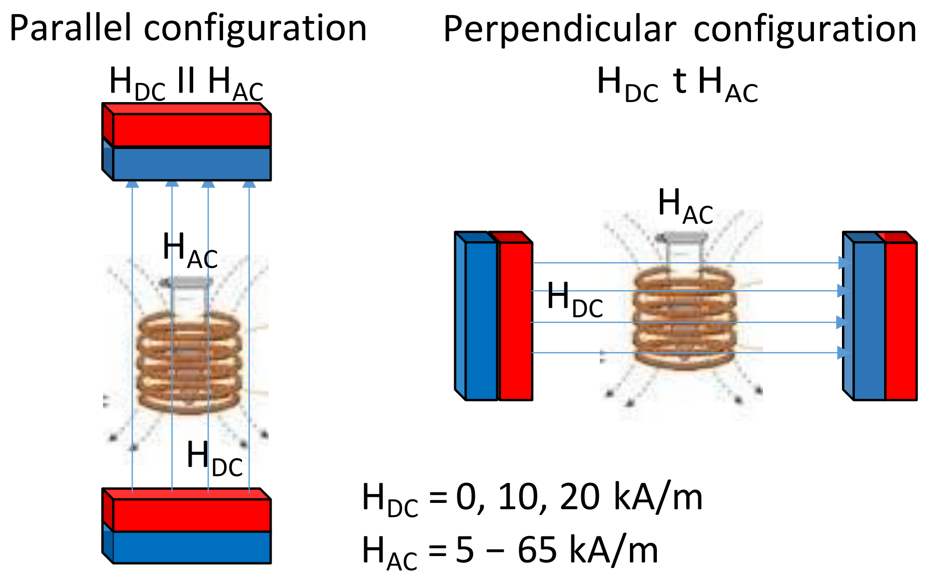

Figure S5. Experimental setup to superpose an H_{DC} over the AMF during MH measurements.

S5. Fitting parameters of the MH data acquired in water and PEG 8K:

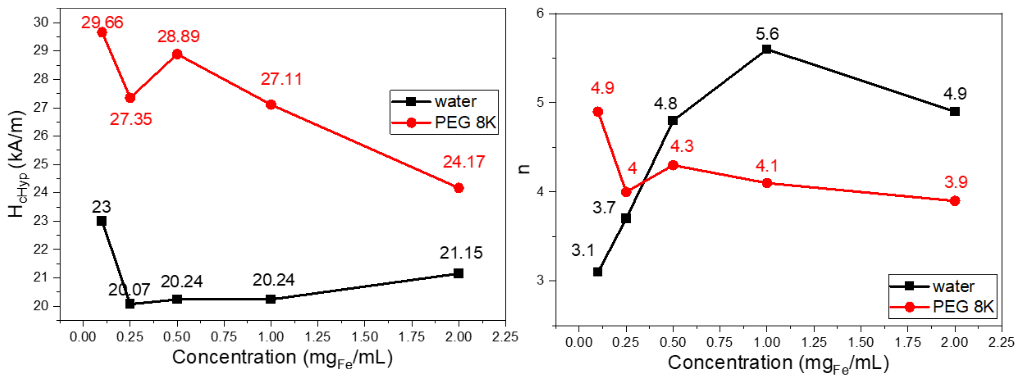

Figure S6. H_{cHyp} (left) and n (right) dependence on the concentration of Zn ferrites for samples measured in water and PEG 8K.

S6. Calculation of the distance between two MNPs uniformly spread in the whole volume:

Assuming that each MNP occupies the same cubic volume with the edge d (which is also de center to center distance between the MNPs), the volume of the cube is:

$$d^3 = V/N$$

where V is the whole volume of the colloid and N is the number of MNPs. N is equal to m/m_0 where m is the mass of all MNPs and m_0 is the mass of a single MNP. m_0 can be expressed from the density and the volume as:

$$m_o = \rho v = \rho\pi D^3/6$$

where D is the diameter of the MNP. After simple algebraic calculation one can obtain:

$$d = D(\pi/6\phi)^{1/3} = D(\rho\pi/6c)^{1/3} = 0.8c^{-1/3}D$$

where ϕ stands for the volume fraction of the MNPs and c is the mass/volume concentration of the colloid.

For the highest concentration used in this study 2 mg_{Fe}/mL, we obtained d = 9.4 D.

S7. Calculation of K_{eff} for samples aligned with the magnetic field and randomly oriented:

For MNPs aligned with the field lines of the external AMF, the following equation was shown to provide the best fit for numerical simulation with AC hysteresis loops [1]:

$$\mu_0 H_{cHyp} M_s = 1.85 K_{eff}\{1 - [\frac{k_B T}{KV} \ln\left(\frac{k_B T}{4\mu_0 H_{cHyp} M_s V f \tau_0}\right)]^{0.8}\} \qquad (S1)$$

while in the case of randomly oriented MNPs the numerical factor is changed as follows:

$$\mu_0 H_{cHyp} M_s = 0.926 K_{eff}\{1 - [\frac{k_B T}{KV} \ln\left(\frac{k_B T}{4\mu_0 H_{cHyp} M_s V f \tau_0}\right)]^{0.8}\} \qquad (S2)$$

The fitting parameters used were: T = 300 K, V = 10^{-23} m^3, f = 355 kHz, $\tau_0 = 10^{-10}$ s and M_S = 380 kA/m.

S8.TEM Images of the MNPs aligned under a DC magnetic field of 10 kA/m

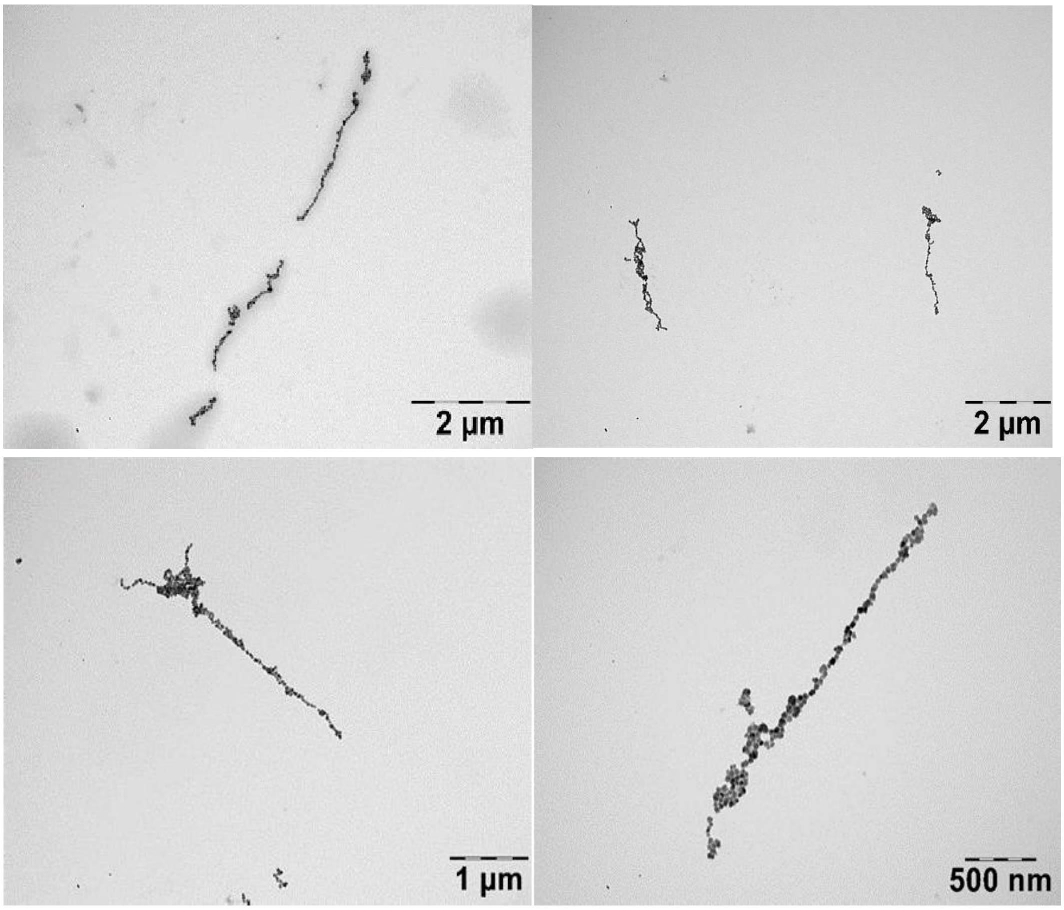

Figure S7. MNPs align themselves in chain under a static DC of 10 kA/m at different magnifications. The samples were disposed on the TEM grid and were allowed to dry under the influence of the DC field.

S9. Fitting parameters for MH data acquired in water with and without H_{DC} of 10 kA/m:

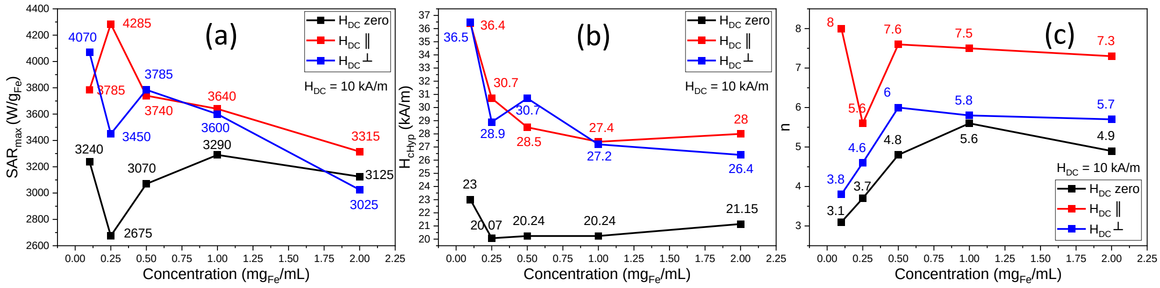

Figure S8. (**a**) The SAR_{max}, (**b**) H_{cHyp}, and (**c**) exponent n dependence on concentration for samples dispersed in water measured with and without an H_{DC} of 10 mT oriented parallel or perpendicular to the AMF lines

S10. Fitting parameters for MH data acquired in water with an H_{DC} of 20 kA/m:

Table S1. SAR_{max}, H_{cHyp}, and n parameters for MH data under an H_{DC} of 20 kA/m.

**Concentration (mg_{Fe}/mL)**	**H_{DC} parallel**			**H_{DC} perpendicular**		
	**SAR_{max} (W/g_{Fe})**	**H_{cHyp} (kA/m)**	**n**	**SAR_{max} (W/g_{Fe})**	**H_{cHyp} (kA/m)**	**n**
0.5	3787	33.8	8.9	2048	39.0	3.9
1	3757	35.0	8.8	1943	38.3	5

S11. Fitting parameters for MH data acquired on samples randomly immobilized in PEG 8K:

Table S2. SAR_{max}, H_{cHyp}, and n for MH data for samples randomly immobilized in PEG 8K and submitted to the AMF and an H_{DC} of 10 kA/m both in parallel and perpendicular configurations.

c (mg_{Fe}/mL)	*H_{DC}*	*H_{cHyp} (kA/m)*	*SAR_{max} (W/g_{Fe})*	*n*
2	0	24.2	1210	3.9
2	II	28.5	1185	4.3
2	⊥	25.2	1125	3.9
1	0	27.1	1480	4.1
1	II	30.5	1410	4.7
1	⊥	26.5	1330	3.9
0.5	0	28.9	1535	4.3
0.5	II	32.3	1520	4.5
0.5	⊥	28.6	1430	4.0
0.25	0	27.4	1040	4.0
0.25	II	31.8	1040	4.5
0.25	⊥	28.5	1025	3.6
0.1	0	29.7	830	4.9
0.1	II	34.5	800	6.3
0.1	⊥	30.9	800	4.0

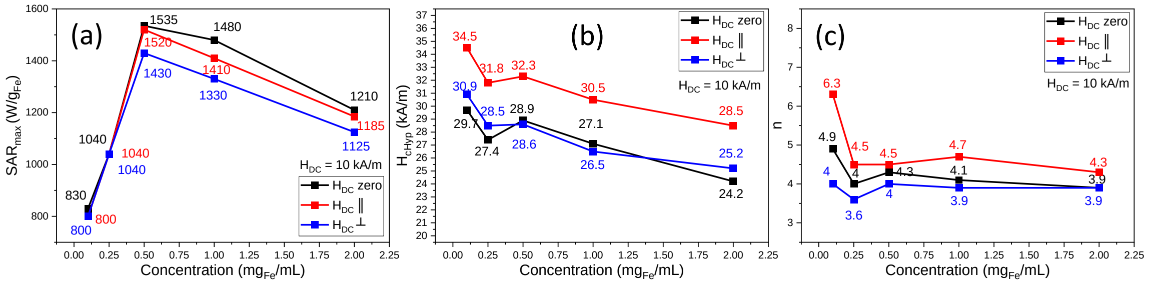

Figure S9. (**a**) The SAR_{max}, (b) H_{cHyp}, and (**c**) exponent *n* dependence on concentration for samples immobilized in solid PEG 8K measured with and without an H_{DC} of 10 mT oriented parallel or perpendicular to the AMF lines.

S12. Influence of the pre-alignment of the MNPs before immobilization on SAR and the effects of the H_{DC} on pre-aligned samples:

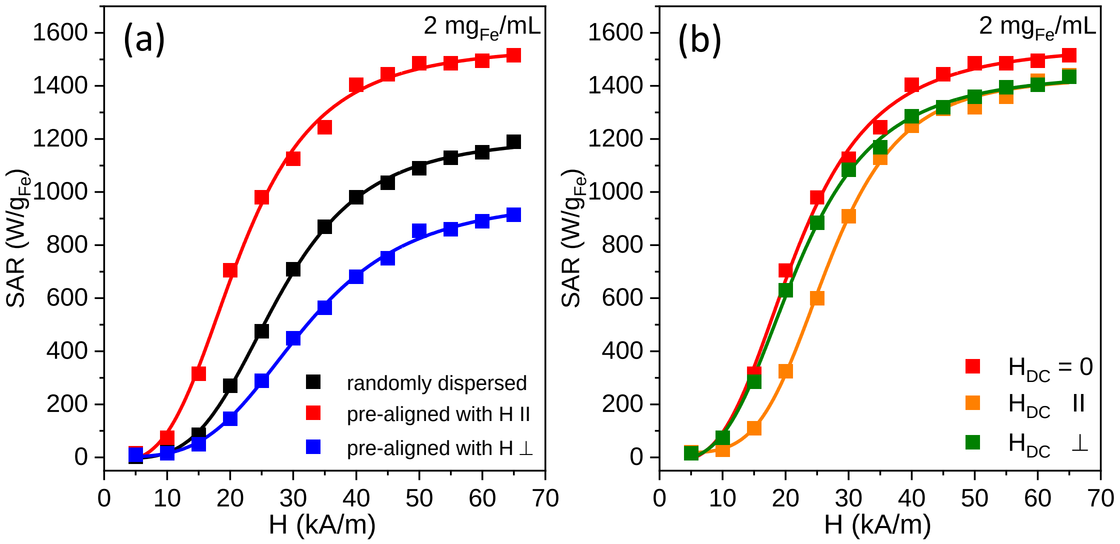

Figure S10. SAR dependence on H for immobilized Zn ferrites in PEG 8K, at a concentration of 2 mg$_{Fe}$/mL, randomly distributed and pre-aligned with an H_{DC} of 65 kA/m either parallel or perpendicular to AMF lines (**a**) and pre-alined with a parallel H_{DC} of 65 kA/m without and with an H_{DC} of 10 kA/m superposed on AMF both in parallel and perpendicular configurations (**b**).

Table S3. SAR_{max}, H_{cHyp}, and n parameters for MH data from Figure S7.

Condition	H_{DC} = 0 kA/m			H_{DC} parallel			H_{DC} perpendicular		
	SAR_{max} (W/g$_{Fe}$)	H_{cHyp} (kA/m)	n	SAR_{max} (W/g$_{Fe}$)	H_{cHyp} (kA/m)	n	SAR_{max} (W/g$_{Fe}$)	H_{cHyp} (kA/m)	n
Pre-aligned with H parallel	1555	17.97	3.34	1450	18.31	3.37	1435	24.27	4.55
Pre-aligned with H perpendicular	980	27.44	3.69						

References:

1. Mehdaoui, B.; Tan, R. P.; Meffre, A.; Carrey, J.; Lachaize, S.; Chaudret, B.; Respaud, M. Increase of magnetic hyperthermia efficiency due to dipolar interactions in low-anisotropy magnetic nanoparticles: Theoretical and experimental results. *Physical Review B* **2013**, *87*, 174419. https://doi.org/10.1103/PhysRevB.87.174419
